# Octupole topological insulating phase protected by a three-dimensional momentum-space nonsymmorphic group

**DOI:** 10.1093/nsr/nwaf137

**Published:** 2025-04-09

**Authors:** Sichang Qiu, Jinbing Hu, Yi Yang, Ce Shang, Shuo Liu, Tie Jun Cui

**Affiliations:** State Key Laboratory of Millimeter Waves, Southeast University, Nanjing 210096, China; College of Optical-Electrical Information and Computer Engineering, University of Shanghai for Science and Technology, Shanghai 200093, China; Department of Physics and HK Institute of Quantum Science and Technology, The University of Hong Kong, Hong Kong 999077, China; Aerospace Information Research Institute, Chinese Academy of Sciences, Beijing 100094, China; State Key Laboratory of Millimeter Waves, Southeast University, Nanjing 210096, China; State Key Laboratory of Millimeter Waves, Southeast University, Nanjing 210096, China

**Keywords:** real projective space, octupole moment, surface-obstructed topological phase, three-dimensional topological circuit

## Abstract

Recent advancements in quantum polarization theory have propelled the exploration of topological insulators (TIs) into the realm of higher-order systems, leading to the study of the celebrated two-dimensional (2D) quadrupole and 3D octupole TIs. Traditionally, these topological phases have been associated with the toroidal topology of the conventional Brillouin zone. This paper reports the discovery of a novel octupole topological insulating phase protected by a 3D momentum-space nonsymmorphic group emerging within the framework of the Brillouin 3D real projective space ($\mathbb {RP}^3$). We theoretically propose the model and its corresponding topological invariant, experimentally construct this insulator within a topological circuit framework and capture the octupole insulating phase as a localized impedance peak at the circuit’s corner. Furthermore, our $\mathbb {RP}^3$ circuit stands out as a pioneering 3D model to simultaneously exhibit both intrinsic, termination-independent symmetry-protected topological phases and extrinsic, termination-dependent surface-obstructed topological phases within the symmetry-protected topological phases. Our results broaden the topological landscape and provide insights into the band theory within the manifold of the Brillouin $\mathbb {RP}^3$ space.

## Teaser

A 3D octupole topological insulator with robust corner states unveiled in topological circuit, demonstrating coexisting intrinsic/extrinsic phases via momentum-space nonsymmorphic symmetry.

## INTRODUCTION

Topological insulators (TIs), remarkable for their robustness against internal defects and external perturbations, have risen to a research prominence in various areas such as photonics [1–5], acoustics [6–10], mechanics [11,12] and electrical circuits [13–25]. The field of topological materials has witnessed significant advancements, ranging from first-order systems to higher-order topological insulators (HOTIs). HOTIs transcend the conventional bulk-edge correspondence theory, featuring boundary states in dimensions lower than $n-1$ [1,2,26–32,32–34]. To date, the topological properties of the aforementioned research are based on the Brillouin zone (BZ) torus $\mathbb {T}^n$ ( $=\underbrace{{\mathbb {S}^1} \times {\mathbb {S}^1} \times \cdots \times {\mathbb {S}^1}}_n$, an orientable *n*-dimensional manifold defined as the product of the bundle of $\mathbb {S}^1$ cylinders), where the Bloch Hamiltonian ${H}(\mathbf {k})$ is restricted to the first Brillouin zone and defined with a reciprocal lattice vector $\mathbf {G}$ as ${H}({\mathbf {k}})={H}({\mathbf {k}+{\bf G}})$ [35–39].

However, the torus is not the only example of a closed compact manifold; the Klein bottle and the real projective plane also belong to this category. Under the $\mathbb {Z}_2$ gauge field [40–43] with the alternative signs of the hopping amplitudes, the symmetries of the system would satisfy projective algebra, extending the Bloch band theory based on the $\mathbb {T}^2$ BZ to the Klein ${\mathbb {K}^2}$ ($={\mathbb {S}^1} \times {\mathbb {X}^1}$ with ${\mathbb {X}^1}$ defining the Möbius bundle) BZ manifold [44,45]. Specifically, the projective symmetry algebra generates an unconventional momentum-space nonsymmorphic ($\mathbf {k}$-NS) symmetry, which contains a fractional translation in the reciprocal lattice. Such phenomena have already been experimentally demonstrated in acoustic crystals in the form of Möbius insulators [43,46]. Recent studies have shown that a real projective plane $\mathbb {RP}^2$ ($={\mathbb {X}^1} \times {\mathbb {X}^1}$) BZ can be employed to construct two-dimensional (2D) HOTIs with quadrupole moments [45,47]. Research has also recently been conducted on the development of HOTIs within $\mathbb {RP}^2$ of real space [48]. The concept is also associated with the half-turn space $\mathbb {HT}^3$ ($={\mathbb {X}^1} \times {\mathbb {S}^1}\times {\mathbb {S}^1}$) [49], which induces surface states of the 3D system. Similarly, the Brillouin Klein space $\mathbb {K}^3$ ($={\mathbb {X}^1} \times {\mathbb {X}^1}\times {\mathbb {S}^1}$) [50] introduces the second pair of twisted boundaries, which further constrains the system and localizes the topological states along 1D hinges as hinge states. The comprehensive understanding of fundamental theory remains incomplete, with the three-dimensional real projective space $\mathbb {RP}^3$ ($={\mathbb {X}^1} \times {\mathbb {X}^1}\times {\mathbb {X}^1}$) representing the elusive final piece of the puzzle in three dimensions. The introduction of the third pair of twisted boundaries may give rise to an intriguing phenomenon, further localizing the topological states at the corners. Moreover, there are two different classifications of HOTIs [51,52]: intrinsic HOTIs, which host symmetry-protected topological phases (SPTPs) induced by bulk gap closures, and extrinsic HOTIs, which host boundary-obstructed topological phases (BOTPs) dependent on boundary termination. To date, the 3D HOTI that simultaneously involves both $\mathbf {k}$-NS symmetries and the coexistence of SPTP and BOTP features has not been reported.

In this paper, we propose a 3D HOTI in the Brillouin $\mathbb {RP}^3$ space, which hosts higher-order corner states induced by the octupole moment of the bulk. Unlike the Benalcazar-Bernevig-Hughes (BBH) model [28,53], which also hosts the bulk octupole moment, we introduce $\mathbf {k}$-NS symmetries along all three axes in momentum space by enforcing the $\mathbb {Z}_2$ gauge field with a chessboard $\pi$-flux configuration to the 3D lattice, transforming the original BZ as a manifold with three pairs of opposing faces glued by a half-twist method [44,47]. These unconventional symmetries divide the 3D BZ into 64 blocks, which are further grouped into eight categories. Selecting one block from each category forms a reduced Brillouin zone that preserves all essential information of the original BZ. Note that these eight blocks should collectively form a closed compact manifold, ensuring that the reduced BZ is a topologically complete and self-contained representation of the system. In particular, the model exhibits both intrinsic and extrinsic HOTI features, where the octupole moment is protected by the $\mathbf {k}$-NS symmetries in the bulk, and edge polarization is induced by either bulk gap closure affecting SPTPs or edge gap closure affecting surface-obstructed topological phases (SOTPs; BOTPs in the 2D case), depending on boundary terminations. We demonstrate the $\mathbb {RP}^3$ HOTI model in a 3D electrical circuit and experimentally observe the octupole corner states by measuring the self-impedance spectra.

## RESULTS

### Brillouin real projective space

**Figure 1.**
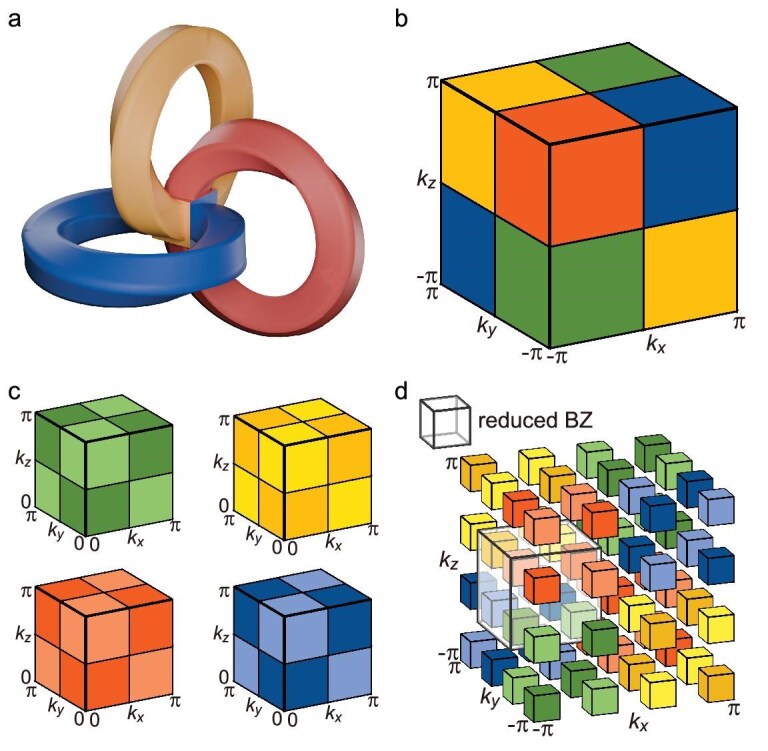
Topological construction of the Brillouin $\mathbb {RP}^3$ space. (a) Schematic illustration depicting the gluing rules of the Brillouin manifold, with a half-twist operation connecting the opposing faces. (b) The $\mathbf {k}$-NS reflection symmetries $\mathcal {M}_{x}$, $\mathcal {M}_{y}$, $\mathcal {M}_{z}$ divide the first BZ into eight segments. (c) The $\mathbf {k}$-NS inversion symmetries $\mathcal {P}_{xy}$, $\mathcal {P}_{xz}$, $\mathcal {P}_{yz}$ further divide one segment in (b) into diagonal and off-diagonal pairs. (d) The $\mathbf {k}$-NS operators $\mathcal {M}_{a}$ and $\mathcal {P}_{ab}$ jointly divide the BZ into 64 blocks, in which a reduced BZ can be defined (semitransparent box) and constructed from any eight uniquely colored blocks.

The $\mathbb {RP}^3$ space is constructed by adhering the opposing faces of a cube with a half twist (Fig. 1a). Mathematically, it is represented as a unit cube ($[0,1] \times [0,1] \times [0,1]$) with each pair of opposing faces identified in the specified relation:


(1) \begin{eqnarray*}
(0, y, z) \sim (1,1-y,1-z), \quad 0 \le y,z \le 1, \\
(x, 0, z) \sim (1-x,1,1-z), \quad 0 \le x,z \le 1, \\
(x, y, 0) \sim (1-x,1-y,1), \quad 0 \le x,y \le 1.\\
\end{eqnarray*}

Following the designated mapping rule, we realize $\mathbb {RP}^3$ in momentum space and derive three $\mathbf {k}$-NS reflection operators for the wave vector $(k_x, k_y, k_z)$, defined as


(2) \begin{eqnarray*}
\mathcal {M}_x:(k_x, k_y, k_z) \rightarrow (-k_x, k_y + \pi , k_z + \pi ), \\
\mathcal {M}_y:(k_x, k_y, k_z) \rightarrow (k_x + \pi , -k_y, k_z + \pi ), \\
\mathcal {M}_z:(k_x, k_y, k_z) \rightarrow (k_x + \pi , k_y + \pi , -k_z), \\
\end{eqnarray*}

where each operator anti-commutes with the others [54–57], such that $\lbrace \mathcal {M}_{a}, \mathcal {M}_{b}\rbrace = 0$ for all $a \ne b$ with $a, b \in \lbrace x, y, z\rbrace$. By applying these operators, the first BZ is divided into eight segments, as shown in Fig. 1b, with diagonally opposing segments forming pairs that are represented with identical colors. Furthermore, the pairwise combination of these three operators induces novel symmetries, leading to the formulation of $\mathbf {k}$-NS inversion operators $\mathcal {P}_{ab} =\mathcal {M}_{a} \mathcal {M}_{b}$:


(3) \begin{eqnarray*}
\mathcal {P}_{xy} :(k_x, k_y, k_z) &\rightarrow (\pi - k_x, \pi - k_y, k_z), \\
\mathcal {P}_{yz} :(k_x, k_y, k_z) &\rightarrow (k_x, \pi - k_y, \pi - k_z), \\
\mathcal {P}_{xz} :(k_x, k_y, k_z) &\rightarrow (\pi - k_x, k_y, \pi - k_z). \\
\end{eqnarray*}

A singular operator $\mathcal {P}_{ab}$ enforces spatial inversion symmetry in the corresponding *a*-*b* plane within the BZ centered at $(\pm \pi /2, \pm \pi /2)$, resulting in the subdivision of each segment into four blocks along diagonal and off-diagonal pairs (Fig. 1c). The application of the remaining two operators yields analogous subdivisions. Consequently, the $\mathbf {k}$-NS symmetric operators $\mathcal {M}$ and $\mathcal {P}$ jointly divide the BZ into 64 blocks, where blocks with the same color denote equivalence in the BZ (Fig. 1d). Therefore, a reduced BZ can be defined and constructed from any eight uniquely colored blocks that are enclosed, for instance, by the semitransparent box in Fig. 1d. The reduced BZ inherits all the topological information from the original BZ, thereby enabling comprehensive analyses of the HOTI, including band-structure properties and topological invariants. The key factor in considering a divided BZ patch as the smallest unit is how it reflects the bulk topological information, including the band structure. Topologically, this requires the BZ patch to be a closed, compact manifold, allowing for the definition of a closed path. This is essential for defining the homotopy group, whose elements correspond to distinct topological phases. We further highlight that, although there are eight fixed points $(\pm \pi /2,\pm \pi /2,\pm \pi /2)$ at the corners of the reduced BZ, they are topologically equivalent to a single point and do not affect the formation of a closed, compact manifold (see the online supplementary material for a detailed discussion).

### Tight-binding model implementation

**Figure 2.**
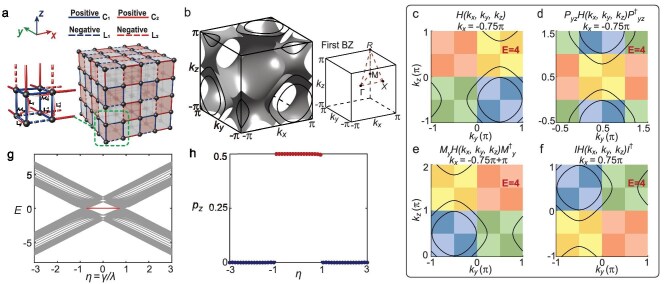
Tight-binding model and the topological properties of the HOTI. (a) The lattice with $2.5\times 2.5\times 2.5$ unit cells, featuring $\mathbf {k}$-NS reflection symmetry $\mathcal {M}_x$. The chessboard $\pi$-flux configuration projectively changes the conventional reflection operator $M_a$ into the $\mathbf {k}$-NS one, which anti-commutes with the translation symmetries along the other two directions $k_b$ and $k_c$. Solid and dashed lines indicate hoppings with positive and negative signs, respectively; blue lines and red lines represent intra-cell and inter-cell couplings, respectively. (b) Iso-energy contour at $E=4$ for $\gamma =1$ and $\lambda =3.3$. Gray contour lines on the $k_{b}$ − $k_{c}$ planes are the projections of the blue contour surface at $k_a=\pm 0.75\pi$. (c) The projection of the iso-energy contour in (b) at the $k_x = -0.75\pi$ cross section. (d–f) The iso-energy contour modified by $\mathbf {k}$-NS symmetry operators $\mathcal {M}_y$, $\mathcal {P}_{yz}$ and the inversion symmetry operator $\mathcal {I}$, respectively. (g) Bulk energy spectrum for a cubic lattice with isotropic coupling strengths and $N_x=N_y=N_z=10$. Corner states are highlighted by red lines. (h) Topological invariants calculated through nested Wilson loops.

To construct the 3D HOTI in the Brillouin $\mathbb {RP}^3$ space, we consider a cubic lattice with eight sites as the unit cell, as shown in Fig. 2a. These eight sites are connected through specially designed hopping connections, with positive (negative) hoppings indicated by solid (dashed) lines fulfilling $\mathbb {Z}_2$ gauge flux. This configuration encloses a $\pi$ flux, resulting in an anti-commutative relation between the $\mathbf {k}$-NS reflection operator $\mathcal {M}_x$ and translation operators $\mathbf {L}_y$, $\mathbf {L}_z$ along the other two directions, respectively. Therefore, in addition to mirror reversion in the $k_x$ direction, $\mathcal {M}_x$ also includes a half-period translation along $k_y$ and $k_z$ simultaneously. Applying these rules to the remaining two directions, we observe a chessboard $\pi$-flux pattern across the *x*-*y, x*-*z* and *y*-*z* planes in Fig. 2a. Note that this model significantly differs from the BBH model [28,53], in which all plaquettes enclose a $\pi$-flux phase.

For convenience, we first assume that the intra-cell coupling strengths $\gamma$ and the inter-cell coupling strengths $\lambda$ are isotropic, that is, $\gamma _{x}=\gamma _{y}=\gamma _{z}=\gamma$ and $\lambda _{x}=\lambda _{y}=\lambda _{z}=\lambda$. The tight-binding (TB) Hamiltonian can be formulated as


(4) \begin{eqnarray*}
&& H(k_x, k_y, k_z) = \lambda (-\!\cos k_x \Gamma ^{\prime }_3 -\sin k_x \Gamma ^{\prime }_0 \\
&& +\, \cos k_y\Gamma ^{\prime }_1 -\sin k_y \Gamma ^{\prime }_2 - \cos k_z \Gamma ^{\prime }_0 \\
&& +\, \sin k_z \Gamma ^{\prime }_5 ) + \gamma \cdot \zeta ,
\end{eqnarray*}

where the $\Gamma ^{\prime }$ matrices are defined as $\Gamma ^{\prime }_0 = \sigma _1 \otimes \Gamma _0$, $\Gamma ^{\prime }_i = \sigma _0 \otimes \Gamma _i\ (i=1,2,3,4)$, $\Gamma ^{\prime }_5 = \sigma _2 \otimes \Gamma _0$, where $\Gamma _0 = \sigma _3\otimes \tau _0$, $\Gamma _j = \sigma _1 \otimes \tau _j$  $(j=1,2,3)$, $\Gamma _4 = \sigma _2 \otimes \tau _0$ and $\zeta = \sigma _3 \tau _1 s_0 + \sigma _1 \tau _0 s_0 - \sigma _3 \tau _2 s_2$, in which $\sigma$, $\tau$ and *s* are Pauli matrices acting on sites along the $x, y, z$ axes, respectively. Constrained by the $\mathbf {k}$-NS symmetry operators, the band structure in the Brillouin $\mathbb {RP}^3$ space displays the corresponding symmetric relations, as evident from the iso-energy contour in Fig. 2b, and panels c–e of Fig. 2 depict the specific effects of the $\mathbf {k}$-NS symmetry operators (see the online supplementary material for a detailed analysis). Thus, the BZ partition in Fig. 1d is further validated. In addition to the $\mathbf {k}$-NS reflection symmetries $\mathcal {M}_a$ and $\mathcal {P}_{ab}$, ${H}(\mathbf {k})$ also retains the conventional inversion symmetry $\mathcal {I}=\mathcal {M}_x\mathcal {M}_y\mathcal {M}_z$ (Fig. 2f), and the chiral symmetry $\mathcal {C} {H}(k_x, k_y, k_z) \mathcal {C}^\dagger = -{H}(k_x, k_y, k_z)$. As shown in Fig. 2g and throughout Fig. 4 below, the energy bands appear in pairs at positive and negative energies due to the chiral symmetry $\mathcal {C}$ of the system. See the online supplementary material for the specific forms of symmetry operators and their effects on the Hamiltonian.

**Figure 3.**
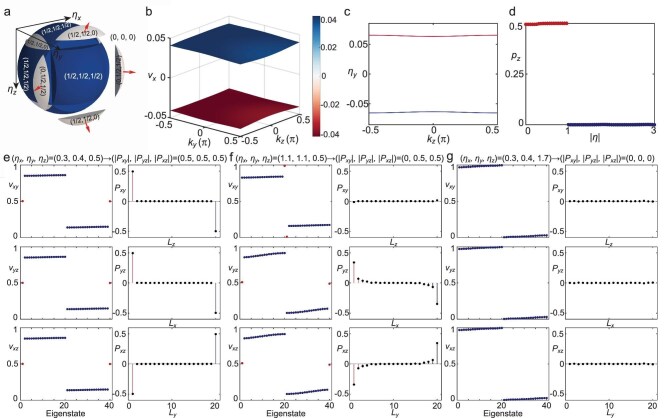
Topological properties of the $\mathbb {RP}^3$ HOTI. (a) Phase diagram of the $\mathbb {RP}^3$ HOTI. (b–d) The procedure for calculating the topological invariant in the reduced BZ involves three rounds of nested Wilson loops along $k_x, k_y, k_z$, respectively ($\gamma =1$). (e–g) The cylindrical geometry with PBCs in the *x* and *y* directions, and OBCs in the *z* direction: Wannier values $v_{ab}^j$ (upper panels) and surface polarization $P_{ab}(L_c)$ (lower panels) in different phase diagram regions. Panels (e) and (f) correspond to regions $(1/2, 1/2, 1/2)$ and $(0, 1/2, 1/2)$ in the phase diagram, respectively, indicating the SOTP transition. Panel (g) corresponds to the region $(0, 0, 0)$, indicating the SPTP transition together with (e).

Figure 2g shows the bulk energy spectrum of the open system as the ratio $\eta =\gamma /\lambda$ varies. Note that this parameter is isotropic in this case. When $|\eta | < 1$, in-gap modes emerge at zero energy (red lines), which indicates the presence of octupole corner states. To better understand and characterize the topological properties of the 3D Brillouin $\mathbb {RP}^3$ model, a topological invariant of 1/2 can be defined using the nested Wilson loop method [47,53], which suggests a nontrivial topological phase for $|\eta | < 1$ and a trivial phase for $|\eta | > 1$ (see Fig. 2h and the online supplementary material). Because of the $\mathbf {k}$-NS symmetry, the topological invariant can also be perfectly defined in the reduced BZ (Fig. 3b–d). See the online supplementary material for a detailed calculation.

### The coexistence of extrinsic and intrinsic HOTI features

Figure 3a presents the phase diagram of edge polarization for the 3D $\mathbb {RP}^3$ HOTI, characterized by surface polarizations $(P_{xy}, P_{yz}, P_{xz})$. A sphere in the parameter space ($\eta _{x}, \eta _{y}, \eta _{z}$) with a radius of $\sqrt{3}$ is divided into two distinct regions, colored blue and gray. Here, $\eta$ represents the ratio of intra-cell to inter-cell hopping strengths along the respective directions. The interior of the sphere represents a topologically nontrivial phase, while the exterior is topologically trivial. By fixing the inter-cell coupling strengths to $\lambda _x=\lambda _y=\lambda _z=1$ and performing band-structure calculations for different $\gamma _x, \gamma _y, \gamma _z$, we find that the gap of bulk bands closes when the sum of the squares of the intra-cell hopping strengths equals that of the inter-cell hopping strengths,


(5) \begin{eqnarray*}
\gamma _x^2+\gamma _y^2+\gamma _z^2=\lambda _x^2+\lambda _y^2+\lambda _z^2=3.
\end{eqnarray*}

To demonstrate the coexistence of the extrinsic and intrinsic HOTI features, we consider a cylindrical geometry with periodic boundary conditions (PBCs) in the *x* and *y* directions, and open boundary conditions (OBCs) in the *z* direction, and calculate the Wannier values $(v_{xy}, v_{yz}, v_{xz})$ and surface polarizations $(P_{xy}, P_{yz}, P_{xz})$ by $P_{ab}=\sum _{r_{c}=1}^{N_{c} / 2} p_{a, b}(r_{c})$, where $p_{a, b}(r_{c})$ is the polarization at each site $r_c$ (see the Methods section below for a detailed calculation) [47,58,59]. We select three points from the parameter space $(\eta _x, \eta _y, \eta _z)$: A (0.3, 0.4, 0.5), B (1.1, 1.1, 0.3) and C (0.3, 0.4, 1.7). Point A belongs to the blue phase region, with surface polarization $(P_{xy}, P_{yz}, P_{xz})=(1/2, 1/2, 1/2)$, indicating a topologically nontrivial phase (Fig. 3e). Point B falls within the gray phase region, with $(P_{xy}, P_{yz}, P_{xz})=(0, 1/2, 1/2)$ (Fig. 3f). The transition from points A to B suggests that increasing the hopping strengths along the periodic directions and crossing the *z* hinges at $|\eta _x|=|\eta _y|=1$ leads to surface gap closure, resulting in the SOTP transition. Panels e–g of Fig. 3 collectively illustrate the SPTP transition: point C lies outside the $\sqrt{3}$ sphere and is classified as intrinsically topologically trivial (Fig. 3g). Thus, transitioning from point A to point C results in bulk gap closure and induces the SPTP transition.

**Figure 4.**
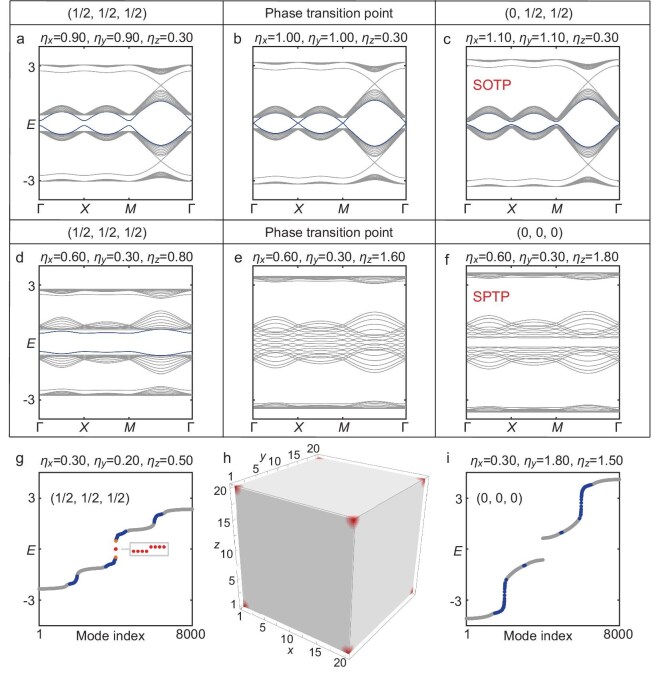
Intrinsic and extrinsic HOTI features of the $\mathbb {RP}^3$ HOTI. (a–c) SOTP transition induced by surface gap closure under PBCs in the *x* and *y* directions, and OBCs in the *z* direction. (d–f) SPTP transition induced by bulk gap closure under the same boundary conditions. (g–i) Phase transition induced by corner gap closure under full OBCs. The appearance of eight corner modes under full OBCs is shown in (h).

We further confirm that edge polarization phase transitions can occur through either bulk or edge gap closures: varying the hopping strengths along the periodic directions results in an edge gap closure, whereas tuning them along the open direction leads to bulk gap closure. With OBCs in the *z* direction and PBCs in the *x* and *y* directions, both surface states (blue lines, in the *y*− *z* plane) and bulk states (gray lines) coexist, as shown in Fig. 4a. Simultaneous changes in hopping strengths along the two periodic directions induce phase transitions; specifically, transitioning from the gray region to the blue region across the hinge at $|\eta _{y}| = |\eta _{z}| = 1$ results in the closure of the surface band gap (Fig. 4b), and the surface states near zero energy vanish when the gap reopens (Fig. 4c). However, adjusting the hopping strengths along the open direction leads to bulk gap closure and an edge phase transition (Fig. 4d–f), resulting in the disappearance of surface states. The bulk band closure induces an SPTP transition, as described in Equation (2), a hallmark of the intrinsic HOTI.

Moreover, these insights into the phase transitions under mixed boundary conditions can be extended to the fully open system. Specifically, crossing the point $(\eta _{x},\eta _{y},\eta _{z})=(\pm 1,\, \pm 1,\, \pm 1)$ from the interior to the exterior of the sphere leads to the disappearance of the corner states (Fig. 4g–i).

### Implementation of the $\mathbb {RP}^3$ topological circuit

The TB model in the quantum electronic system can be directly implemented in the electric circuit by mapping the TB Hamiltonian in Equation (4) onto the circuit Laplacian. We realize the $\mathbb {Z}_2$ gauge connections in circuits by utilizing the opposite phases of the admittance in capacitors and inductors. Two pairs of capacitors and inductors $(C_1, L_1)$ and $(C_2=\lambda C_1, L_1=\lambda L_2)$ are employed as the intra-cell and inter-cell couplings in the circuit, respectively, as shown in Fig. 3a. Note that the boundary circuit nodes should be grounded with additional capacitors and inductors to maintain the same resonant frequency as the bulk nodes, $\omega _0 = 1 / \sqrt{L_1 C_1} = 1 / \sqrt{L_2 C_2}$ (see the online supplementary material). In this work, we specify $C_1=1\, \text{nF}$, $C_2=3.3\, \text{nF}$, $L_1=3.3\,\mu \text{H}$, $L_2=1\, \mu \text{H}$.

According to Kirchhoff’s current law, we can derive the circuit Laplacian that characterizes the behavior of the circuit as $\mathbf {J}(\omega ) = i\omega \mathbf {C} -i/\omega \mathbf {W}$, where $\mathbf {C}$ and $\mathbf {W}$ are the matrices of capacitance and inverse inductance, respectively. Note that, as the admittance of the capacitor and inductor cancel at $\omega _0$, the diagonal terms of $\mathbf {J}(\omega )$ vanish at $\omega _0$. Consequently, $\mathbf {J}(\omega _0)$ takes exactly the form of the Hamiltonian of the quantum electronic system in Equation (4), up to a scaling factor of $i \sqrt{C_1/L_1}$. One can obtain the eigenfrequencies of the circuit by using the dynamical matrix ${\mathbf { D}} = {\mathbf { C}}^{-{1}/{2}}{\mathbf { W}} {\mathbf { C}}^{-{1}/{2}}$ [16,60].

**Figure 5.**
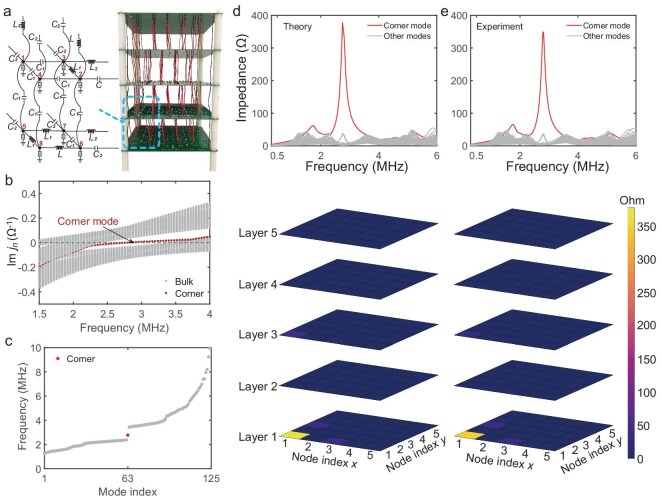
Topological properties of the circuit realization. (a) Circuit diagram of the unit cell and the experimental sample. The grounded terms ‘z’ represent shunt resonant circuits with capacitors and inductors connected in parallel. (b) Eigenvalue spectrum of $J(\omega )$. (c) Eigenfrequency of the finite circuit characterized by the ${D}$ matrix. The corner mode is highlighted by the red dot. (d) Theoretical calculated impedance spectra (upper panel) and the impedance distribution at resonant frequency 2.77 MHz (lower panel). (e) Experimental impedance spectra measured by the vector network analyzer (upper panel) and the impedance distribution at 2.77 MHz (lower panel). Both theoretical and experimental results clearly demonstrate the presence of the octupole corner state localized at the bottom left corner.

To experimentally demonstrate the octupole corner state induced by the 3D Brillouin $\mathbb {RP}^3$ model, we fabricated a 3D circuit with $2.5\times 2.5\times 2.5$ unit cells by connecting five layers of printed circuit boards via copper wires (Fig. 5a). Low dc resistance inductors with a maximum tolerance of 5% were selected for the experiment to improve the quality factor of the circuit while maintaining sufficient precision. In the eigenvalue spectrum of the circuit Laplacian (Fig. 5b), the frequencies satisfying $\mathbf {J}(\omega )=0$ represent the eigenfrequencies of the circuit system, as indicated by the intersections of the eigenvalue spectrum with the gray dashed line. This can be observed from the eigenfrequencies of the finite circuit in Fig. 5c, where an in-gap mode at the resonant frequency $\omega _0=2.77\,\rm {MHz}$ signifies the presence of the octupole corner state. It has been suggested that the eigenstates of the circuit can be accessed by measuring the self-impedance across all circuit nodes at $\omega _0$, which is proportional to the square of the eigenstates in the TB Hamiltonian [61]. In the experiment, we obtained the self-impedance spectra by measuring the circuit’s scattering parameters using a vector network analyzer (Tektronix TTR506A). As depicted in the upper panel of Fig. 5d and e, the self-impedance spectra obtained at all circuit nodes (upper panel) exhibit high consistency between theoretical and experimental results. The spectrum measured at the corner node reveals a prominent peak at 2.77 MHz (red curve), signifying the presence of the topological corner state. This is further validated by the impedance distribution at the corner mode frequency of 2.77 MHz across all circuit nodes, as shown in the lower panel of Fig. 5d and e. Note that, due to the configuration with a half-integer number of unit cells in all three dimensions, the current circuit supports only one corner state, with an impedance peak localized at a single corner (Fig. 5d and e). Additionally, the corner state is equally localized in all spatial directions. The apparent localization in the *z* direction in the figure is simply for illustration, as the states are shown as slices along the *z* axis. The results of the 3D $\mathbb {RP}^3$ topological circuit with an integer number of unit cells are given in the online supplementary material. We also verify the phase transitions among the bulk, surface, hinge and corner states of our 3D HOTI in the circuit system by calculating the band structure and eigenstates of the circuit in both fully open and semi-open scenarios, which align with the results from the electronic system shown in Fig. 4 (see the online supplementary material).

## CONCLUSIONS

In conclusion, we experimentally demonstrate a novel octupole topological insulating phase induced by the 3D real projective space $\mathbb {RP}^3$ in momentum space. A $\pi$-flux chessboard pattern enforced by the $\mathbf {k}$-NS symmetries under the $\mathbb {Z}_2$ gauge field is shown to give rise to the unconventional features of the 3D $\mathbb {RP}^3$ HOTI, distinguishing it from the first HOTI with all plaquettes enclosed by a $\pi$-flux [28,53]. Note that the current method for implementing the $\mathbb {Z}_2$ gauge field in electrical circuits with inductors and capacitors allows the direct mapping of the Hamiltonian in the quantum electronic system only at the resonant frequency. This prevents us from measuring the eigenstates of the other modes (e.g. surface and hinge states). Alternative approaches for realizing the negative coupling include the use of negative capacitors with negative impedance convertor [62], or employing a pair of circuit nodes with twist connection [63]. Both methods enable access to all eigenstates.

## METHODS

### Topological invariant calculated in the reduced BZ

As shown in Fig. 3b–d, the topological invariant of the $\mathbb {RP}^3$ HOTI model can be effectively calculated in the reduced BZ, where $k_x \in (-\pi , 0)$, $k_y \in ( -0.5 {\pi }, 0.5 {\pi } )$, $k_z \in ( -0.5 {\pi }, 0.5 {\pi } )$. Under the constraints of the $\mathbf {k}$-NS reflection symmetries, the eigenstates used to calculate the first-round nested Wilson loop will be modified as


(6) \begin{eqnarray*}
|u_{\mathbf {k}}^n \rangle _{\mathcal {M}_x}={\mathcal {M}_x}|u_{\mathbf {k}}^n \rangle .
\end{eqnarray*}

Similarly, the recombined eigenstates $\mathinner {|{w_{x,\mathbf {k}}^{+,j}}\rangle }=\sum _{n=1}^{N_{\text{occ}}} \mathinner {|{u_\mathbf {k}^n}\rangle } [{v_{x,\mathbf {k}}^{+,j}}]^n$ used to calculated the second-round nested Wilson loop will be modified as


(7) \begin{eqnarray*}
\mathinner {|{w_{x,\mathbf {k}}^{+,j}}\rangle }_{\mathcal {M}_y} = \mathcal {M}_y\sum _{n=1}^{N_{\text{occ}}} \mathinner {|{u_\mathbf {k}^n}\rangle } [{v_{x,\mathbf {k}}^{+,j}}]^n,
\end{eqnarray*}

and the line elements of the third-round nested Wilson loop will be modified as


(8) \begin{eqnarray*}
\mathinner {|{w_{y,\mathbf {k}}^{{+_x},{+_y}}}\rangle }_{\mathcal {M}_z} = \mathcal {M}_z\sum _{n=1}^{N_{\text{occ}}} \mathinner {|{u_{x,\mathbf {k}}^{\pm ,n}}\rangle } [{\eta _{y,\mathbf {k}}^{{+_x},\pm }}]^n.
\end{eqnarray*}

Thus, by repeating the nested Wilson loop calculation steps in the online supplementary material, while restricting the integration range to the reduced BZ, we can obtain the results of nested Wilson loops. By comparing Fig. 2h and Fig. 3d, we confirm that the reduced BZ can be effectively used to calculate the topological invariant of the $\mathbb {RP}^3$ HOTI.

### Edge polarizations

To calculate surface polarization of the $\mathbb {RP}^3$ HOTI and confirm the coexistence of SOTPs and SPTPs [45,47,59], a *z*-open cylinder geometry with $N_x \times N_y \times N_z$ sites is considered. The surface polarization $P_{xy}$ can be obtained by the following methods.

1. Treat the *z*-open cylinder as a wide pseudo-2D structure by absorbing the labels $r_z \in 1, \ldots , N_z$ into the supercell lattice degrees of freedom, since there is no crystal momenta $k_z$.
2. Solve the eigenvectors $\mathinner {|{u}\rangle }_{k_x, k_y}^n$ of the pseudo-2D *k*-space Hamiltonian, and first perform the Wilson loop $W_{x}$ along $k_x$ to obtain its eigenstates $\mathinner {|{v}\rangle }_{x,k_y}^j$.
3. Construct the Wannier states $\mathinner {|{v_{y,k_x,k_y}^{+,j}}\rangle } = \sum _{n=1}^{N_{\text{occ}}} \mathinner {|{u_{k_x, k_y}^n}\rangle } [{v_{x,k_y}^{+,j}}]^n$ and use them to calculate the nested Wilson loop $\tilde{W}^{+x}_{y}$ along $k_y$ (see the online supplementary material). This allows us to obtain the eigenstates $\mathinner {|{\eta }\rangle }_{k_x, k_y}^n$. We can now calculate the density of the hybrid Wannier function using the equation
  (9) \begin{eqnarray*}
\rho ^j(r_z)=\frac{1}{N_xN_y}\sum _{k_x,k_y,\alpha } \bigg |\sum _{n}[v^n_{k_x,k_y}]^{r_z,\alpha }[\eta ^j_{k_x,k_y}]^n\bigg |^2,
\end{eqnarray*}
  where $[v^n_{k_x,k_y}]_{r_z,\alpha }$ is the corresponding component of the *n*th Wannier state, and $[\eta ^j_{k_x,k_y}]^n$ is the *n*th component of the *j*th eigenstate $\mathinner {|{\eta }\rangle }_{k_x, k_y}^n$. The polarization at each site $r_z$ is given by
  (10) \begin{eqnarray*}
p_{x,y}(r_z)=\sum _{j}\rho ^j(r_z)\eta _{x,y}^j.
\end{eqnarray*}
4. Finally, calculate the surface polarization by summing $p_{x,y}(r_z)$ over half of the system along *z*:
  (11) \begin{eqnarray*}
p_{x,y}^{\rm {surface}}=\sum _{r_z=1}^{N_z/2}p_{x,y}(r_z).
\end{eqnarray*}

### Simulation and experiment

The Agilent Design System software is employed for the numerical simulation of a circuit of $5\times 5\times 5$ unit cells, using the exact values of the components in the fabricated sample. The sample consists of five layers of PBCs, with each adjacent layer connected by high-temperature Teflon wires. To minimize experimental deviations, the maximal tolerance for circuit components is capped at $5\%$, and inductors are selected with minimal direct current resistance.

The eigenstate of the circuit system can be accessed through the self-impedance of each circuit node [61], as demonstrated below. According to Kirchhoff’s circuit laws in the frequency domain, eigenstate $\mathbf {V}$ and eigenfrequencies $\omega$ of the circuit can be obtained by solving the eigenvalue problem


(12) \begin{eqnarray*}
H_J\mathbf {V}=\lambda \mathbf {V},
\end{eqnarray*}

where $H_J$ takes exactly the form of the Hamiltonian of the TB model, with eigenvalue $\lambda =(W_t-\omega ^2 C_t)/(\omega ^2 C)$. The self-impedance of each circuit node is defined as


(13) \begin{eqnarray*}
Z_{aa}(\omega ) &=& \frac{V_a}{I_a} = \frac{1}{i\omega C} \bigg ( \frac{1}{H_J - \lambda } \bigg )_{aa} \\
&=& \frac{1}{i\omega C} \sum _{n} \frac{|V_n^a|^2}{\lambda _n - \lambda }.
\end{eqnarray*}

Equation (13) indicates that, when $\lambda$ equals the *n*th mode of $H_J$, the denominator becomes zero, resulting in divergences of the expression. For example, the impedance spectrum measured at the corner nodes is expected to show a prominent peak at the corresponding frequency, indicating the presence of the corner state. This suggests that $Z_{aa}(\omega )$ scanned across all circuit nodes at the *n*th eigenfrequency $\omega _n$ represents the squared magnitudes $|V_n|^2$ of the eigenstates associated with the *n*th mode of $H_J$ up to a scaling factor of $1 / i\omega C$.

In the circuit experiment, we employed the vector network analyzer (Tektronix TTR506A) to measure the $S_{11}$ parameters of each node. The reflection coefficient $S_{11}$ of the circuit can be transformed into the self-impedance using the formula


(14) \begin{eqnarray*}
Z_{11} = Z_0 \frac{1 + S_{11}}{1 - S_{11}},
\end{eqnarray*}

where $Z_0$ represents the characteristic impedance.

## Supplementary Material

nwaf137_Supplemental_File
