# Supplementary material for: Octupole topological insulating phase protected by a three-dimensional momentum-space nonsymmorphic group
Source: Natl Sci Rev. 2025 Apr 9;12(7):nwaf137. doi: 10.1093/nsr/nwaf137 (PMC12153757; doi:10.1093/nsr/nwaf137)
Supplement: nwaf137_Supplemental_File [file nwaf137_supplemental_file.pdf]

Supplementary Material for
Octupole topological insulating phase protected by three-dimensional momentum-space nonsymmorphic group

Sichang Qiu,[1,*] Jinbing Hu,[2,*] Yi Yang,[3] Ce Shang,[4,†] Shuo Liu,[1,‡] and Tie Jun Cui[1,§]

[1]*State Key Laboratory of Millimeter Waves,*
Southeast University, Nanjing 210096, China.
[2]*College of Optical-Electrical Information and Computer Engineering,*
University of Shanghai for Science and Technology, Shanghai 200093, China
[3]*Department of Physics and HK Institute of Quantum Science and Technology,*
The University of Hong Kong, Pokfulam, Hong Kong, China
[4]*Aerospace Information Research Institute,*
Chinese Academy of Sciences, Beijing 100094, China
(Dated: April 2, 2025)

In this supplementary material, we discuss (I) the proof that $\mathbb{RP}^3$ is a closed, compact manifold; (II) symmetries in the $\mathbb{RP}^3$ HOTI; (III) topological invariants calculated by nested Wilson loops; (IV) topological invariants with symmetry constraint; (V) parameter tuning in semi-open circuit systems; (VI) topology of the finite circuit with a complete period; (VII) details for the experimental circuit.

Supplementary Note 1. The proof that $\mathbb{RP}^3$ is a closed, compact manifold

It is common for a point to be fixed under the actions of two or more symmetries. For instance, in the 2D case, the point $(\pi/2, \pi/2)$ is mapped to $(-\pi/2, \pi/2)$ by the C_4 symmetry, and then back to $(\pi/2, \pi/2)$ by the mirror symmetry along the x-axis. The critical aspect of whether a divided BZ patch can be considered as the smallest unit is how this BZ patch reflects the bulk topological information, including the band structure; topologically, this is equivalent to the requirement that the BZ patch must be a closed, compact manifold, so that a closed path can be defined. This is a prerequisite for defining the homotopy group, whose elements correspond to distinct topological phases of the system's bulk. From this perspective, among the conventional symmetries, only translation symmetry can sew together the perimeter of the BZ, forming a compact BZ manifold,

* These authors contributed equally
† shangce@aircas.ac.cn
‡ liushuo.china@seu.edu.cn
§ tjcui@seu.edu.cn

denoted $\mathbb{T}^d$ (where d is the system dimension). The recently proposed momentum-space glide reflection (denoted as **k**-NS $\mathcal{M}_x$) has been shown to possess this sewing ability. For example, the combination of **k**-NS $\mathcal{M}_x$ and translation $\mathbf{L}_y$ generates a non-orientable Klein bottle BZ[1]. Similarly, the $\mathbb{RP}^3$ in our study is generated by three momentum-space glide reflections: **k**-NS $\mathcal{M}_x, \mathcal{M}_y, \mathcal{M}_z$.

Here, we want to emphasize that, although there are eight points $(\pm\pi/2, \pm\pi/2, \pm\pi/2)$ fixed at the corners of the reduced BZ, these points are topologically identified as a single point. Therefore, they do not interfere with the formation of a closed, compact manifold. To illustrate this more clearly, we present several examples of closed, compact manifolds that can be constructed. Figure S1 displays these basic 2D closed manifolds—$\mathbb{T}^2, \mathbb{K}^2$, and $\mathbb{RP}^2$—along with their corresponding topologically equivalent rectangles, showing the mapping between the four points (in blue) of the rectangles and a single point on the manifold.

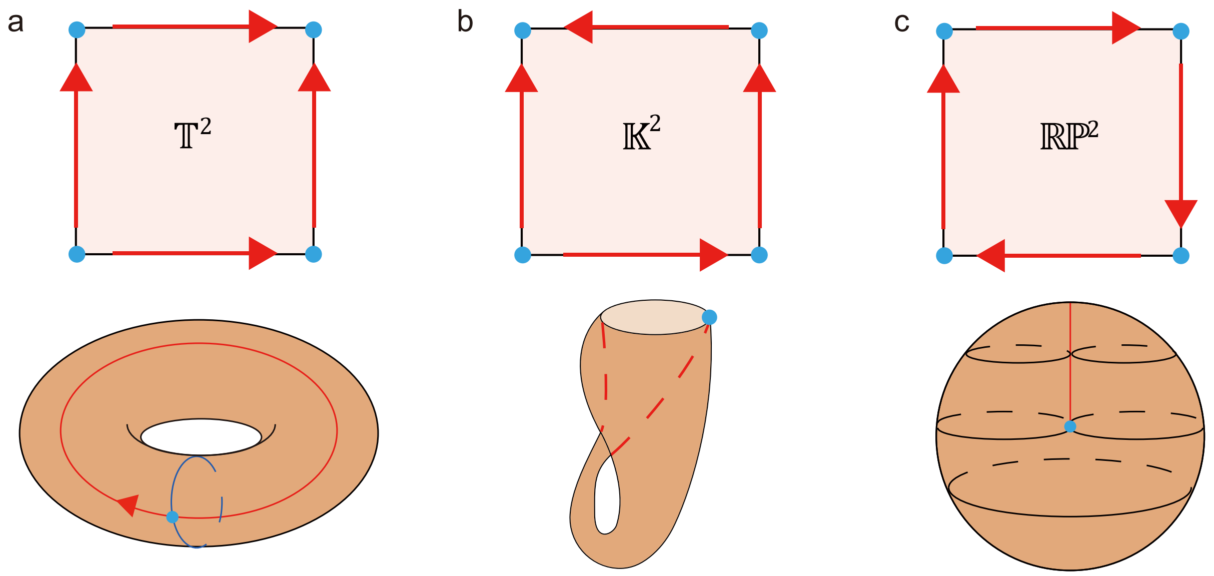

Supplementary Figure S1. Three types of 2D closed manifolds. (a) $\mathbb{T}^2$ BZ manifold. (b) $\mathbb{K}^2$ BZ manifold. (c) $\mathbb{RP}^2$ BZ manifold.

When extending to 3D closed manifolds, the concept is similarly applied to half-turn space $\mathbb{HT}^3$, Klein space $\mathbb{K}^3$ and our $\mathbb{RP}^3$ model. The corresponding gluing rules for these 3D manifolds are illustrated in Figure S2. In the 3D case, the eight points located at the corners of the cube are topologically equivalent, corresponding to a single point in the BZ manifold. The $\mathbb{RP}^3$ model effectively captures the complete topological information within the reduced Brillouin zone. This capability is further validated through the calculation of topological invariants in the reduced

Brillouin zone, as shown in Figures 3(b-d) of the main text.

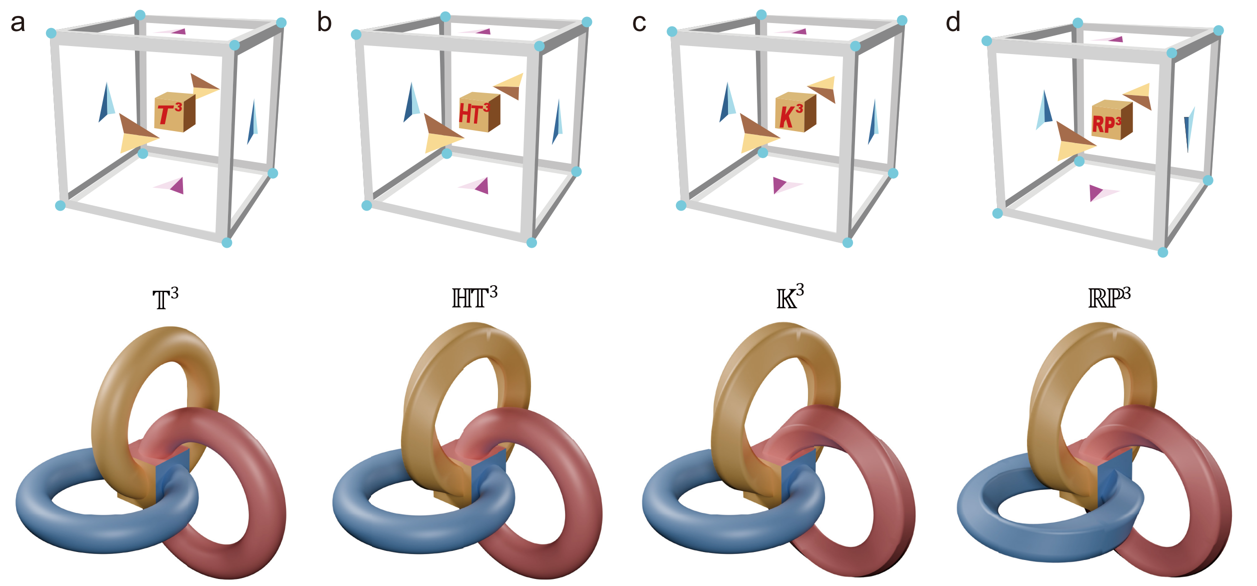

Supplementary Figure S2. Four types of 3D closed manifolds. The direction and color of the arrows denote the gluing rules. (a) $\mathbb{T}^3$ BZ manifold. (b) $\mathbb{HT}^3$ BZ manifold. (c) $\mathbb{K}^3$ BZ manifold. (d) $\mathbb{RP}^3$ BZ manifold.

Supplementary Note 2. Symmetries in the $\mathbb{RP}^3$ HOTI

In order to study the intrinsic symmetries of the $\mathbb{RP}^3$ HOTI, we set the intra-cell hopping strength γ to 1 and transform the real-space tight-binding model into the momentum-space form by the Fourier transformation, yielding the following Hamiltonian under PBCs,

$$H(k_x,k_y,k_z)=\begin{bmatrix} 0 & 0 & 1-\lambda e^{-ikx} & 1+\lambda e^{iky} & 1-\lambda e^{ikz} & 0 & 0 & 0 \\ 0 & 0 & -1+\lambda e^{-iky} & 1+\lambda e^{ikx} & 0 & 1-\lambda e^{ikz} & 0 & 0 \\ 1-\lambda e^{ikx} & -1+\lambda e^{iky} & 0 & 0 & 0 & 0 & 1+\lambda e^{ikz} & 0 \\ 1+\lambda e^{-iky} & 1+\lambda e^{-ikx} & 0 & 0 & 0 & 0 & 0 & 1+\lambda e^{ikz} \\ 1-\lambda e^{-ikz} & 0 & 0 & 0 & 0 & 0 & -1-\lambda e^{-ikx} & -1+\lambda e^{iky} \\ 0 & 1-\lambda e^{-ikz} & 0 & 0 & 0 & 0 & 1+\lambda e^{-iky} & -1+\lambda e^{ikx} \\ 0 & 0 & 1+\lambda e^{-ikz} & 0 & -1-\lambda e^{ikx} & 1+\lambda e^{iky} & 0 & 0 \\ 0 & 0 & 0 & 1+\lambda e^{-ikz} & -1+\lambda e^{-iky} & -1+\lambda e^{-ikx} & 0 & 0 \end{bmatrix}. \tag{1}$$

Hamiltonian (1) follows

$$\begin{aligned}\mathcal{M}_x H(k_x,k_y,k_z)\mathcal{M}_x^\dagger &= H(-k_x,\pi+k_y,\pi+k_z),\\ \mathcal{M}_y H(k_x,k_y,k_z)\mathcal{M}_y^\dagger &= H(\pi+k_x,-k_y,\pi+k_z),\\ \mathcal{M}_z H(k_x,k_y,k_z)\mathcal{M}_z^\dagger &= H(\pi+k_x,\pi+k_y,-k_z),\end{aligned} \tag{2}$$

and

$$\begin{aligned}\mathcal{P}_{xy} H(k_x,k_y,k_z)\mathcal{P}_{xy}^\dagger &= H(\pi-k_x,\pi-k_y,k_z),\\ \mathcal{P}_{xz} H(k_x,k_y,k_z),\mathcal{P}_{xz}^\dagger &= H(\pi-k_x,k_y,\pi-k_z)\\ \mathcal{P}_{yz} H(k_x,k_y,k_z)\mathcal{P}_{yz}^\dagger &= H(k_x,\pi-k_y,\pi-k_z).\end{aligned} \tag{3}$$

The above symmetry operators take the following forms in our 3D HOTI: $\mathcal{M}_x = \sigma_0 \otimes \tau_1 \otimes \xi_3$, $\mathcal{M}_y = \sigma_0 \otimes \tau_1 \otimes \xi_1$, $\mathcal{M}_z = \sigma_1 \otimes \tau_3 \otimes \xi_0$; $\mathcal{P}_{xy} = \mathcal{M}_x\mathcal{M}_y$, $\mathcal{P}_{xz} = \mathcal{M}_x\mathcal{M}_z$, $\mathcal{P}_{yz} = \mathcal{M}_y\mathcal{M}_z$, respectively.

The incorporation of synthetic gauge fields endows $H(k_x,k_y,k_z)$ with the following intrinsic **k**-NS symmetries. Eq. (4) describes the symmetries exhibited by the Hamiltonian under the action of the **k**-NS reflection operators $\mathcal{M}_i$ $(i = x, y, z)$, which can be collectively referred as group $\mathcal{M}$,

$$\begin{aligned}\mathcal{M}_x H(k_x,k_y,k_z)\mathcal{M}_x^\dagger &= H(-k_x,\pi+k_y,\pi+k_z),\\ \mathcal{M}_y H(k_x,k_y,k_z)\mathcal{M}_y^\dagger &= H(\pi+k_x,-k_y,\pi+k_z),\\ \mathcal{M}_z H(k_x,k_y,k_z)\mathcal{M}_z^\dagger &= H(\pi+k_x,\pi+k_y,-k_z),\end{aligned} \tag{4}$$

and the precise form of the **k**-NS reflection operators can be represented in matrices,

$$\mathcal{M}_x = \begin{bmatrix} 0&0&1&0&0&0&0&0\\ 0&0&0&-1&0&0&0&0\\ 1&0&0&0&0&0&0&0\\ 0&-1&0&0&0&0&0&0\\ 0&0&0&0&0&0&1&0\\ 0&0&0&0&0&0&0&-1\\ 0&0&0&0&1&0&0&0\\ 0&0&0&0&0&-1&0&0 \end{bmatrix}, \quad \mathcal{M}_y = \begin{bmatrix} 0&0&0&1&0&0&0&0\\ 0&0&1&0&0&0&0&0\\ 0&1&0&0&0&0&0&0\\ 1&0&0&0&0&0&0&0\\ 0&0&0&0&0&0&0&1\\ 0&0&0&0&0&0&1&0\\ 0&0&0&0&0&1&0&0\\ 0&0&0&0&1&0&0&0 \end{bmatrix}, \quad \mathcal{M}_z = \begin{bmatrix} 0&0&0&0&1&0&0&0\\ 0&0&0&0&0&1&0&0\\ 0&0&0&0&0&0&-1&0\\ 0&0&0&0&0&0&0&-1\\ 1&0&0&0&0&0&0&0\\ 0&1&0&0&0&0&0&0\\ 0&0&-1&0&0&0&0&0\\ 0&0&0&-1&0&0&0&0 \end{bmatrix}. \tag{5}$$

The group $\mathcal{P}$ operators are generated under the pairwise action of the group $\mathcal{M}$, and force the Hamiltonian to be inversion-symmetric about the point $(\pi/2, \pi/2)$ on a certain plane,

$$\begin{aligned}\mathcal{P}_{xy} H(k_x,k_y,k_z)\mathcal{P}_{xy}^\dagger &= H(\pi-k_x,\pi-k_y,k_z),\\ \mathcal{P}_{xz} H(k_x,k_y,k_z)\mathcal{P}_{xz}^\dagger &= H(\pi-k_x,k_y,\pi-k_z)\ ,\\ \mathcal{P}_{yz} H(k_x,k_y,k_z)\mathcal{P}_{yz}^\dagger &= H(k_x,\pi-k_y,\pi-k_z),\end{aligned} \tag{6}$$

with the detailed forms defined as

$$\mathcal{P}_{xy}=\begin{bmatrix}0&1&0&0&0&0&0&0\\-1&0&0&0&0&0&0&0\\0&0&0&1&0&0&0&0\\0&0&-1&0&0&0&0&0\\0&0&0&0&0&1&0&0\\0&0&0&0&-1&0&0&0\\0&0&0&0&0&0&0&1\\0&0&0&0&0&0&-1&0\end{bmatrix},\quad \mathcal{P}_{xz}=\begin{bmatrix}0&0&0&0&0&0&-1&0\\0&0&0&0&0&0&0&1\\0&0&0&0&1&0&0&0\\0&0&0&0&0&-1&0&0\\0&0&-1&0&0&0&0&0\\0&0&0&1&0&0&0&0\\1&0&0&0&0&0&0&0\\0&-1&0&0&0&0&0&0\end{bmatrix},\quad \mathcal{P}_{yz}=\begin{bmatrix}0&0&0&0&0&0&0&-1\\0&0&0&0&0&0&-1&0\\0&0&0&0&0&1&0&0\\0&0&0&0&1&0&0&0\\0&0&0&-1&0&0&0&0\\0&0&-1&0&0&0&0&0\\0&1&0&0&0&0&0&0\\1&0&0&0&0&0&0&0\end{bmatrix}. \tag{7}$$

Furthermore, the $\mathbb{RP}^3$ HOTI also hosts the conventional inversion symmetry

$$\mathcal{I}H(k_x,k_y,k_z)\mathcal{I}^\dagger=H(-k_x,-k_y,-k_z), \tag{8}$$

where

$$\mathcal{I}=\begin{bmatrix}0&0&0&0&0&1&0&0\\0&0&0&0&-1&0&0&0\\0&0&0&0&0&0&0&-1\\0&0&0&0&0&0&1&0\\0&1&0&0&0&0&0&0\\-1&0&0&0&0&0&0&0\\0&0&0&-1&0&0&0&0\\0&0&1&0&0&0&0&0\end{bmatrix}, \tag{9}$$

and the chiral symmetry

$$\mathcal{C}H(k_x,k_y,k_z)\mathcal{C}^\dagger=-H(k_x,k_y,k_z), \tag{10}$$

where

$$\mathcal{C}=\sigma_3\otimes\tau_3\otimes\xi_0=\begin{bmatrix}1&0&0&0&0&0&0&0\\0&1&0&0&0&0&0&0\\0&0&-1&0&0&0&0&0\\0&0&0&-1&0&0&0&0\\0&0&0&0&-1&0&0&0\\0&0&0&0&0&-1&0&0\\0&0&0&0&0&0&1&0\\0&0&0&0&0&0&0&1\end{bmatrix}. \tag{11}$$

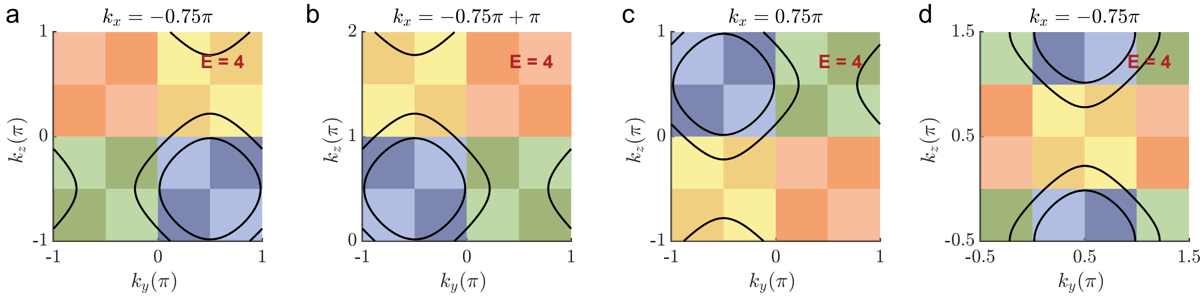

Supplementary Figure S3. Iso-energy contours at E=4 on the $y - z$ plane. **a** $k_x = -0.75\pi$, $(k_y, k_z) \in [-\pi, \pi) \times [-\pi, \pi)$. **b** $k_x = -0.75\pi + \pi$, $(k_y, k_z) \in [-\pi, \pi) \times [0, 2\pi)$. **c** $k_x = 0.75\pi$, $(k_y, k_z) \in [-\pi, \pi) \times [-\pi, \pi)$. **d** $k_x = -0.75\pi$, $(k_y, k_z) \in [-0.5\pi, 1.5\pi) \times [-0.5\pi, 1.5\pi)$.

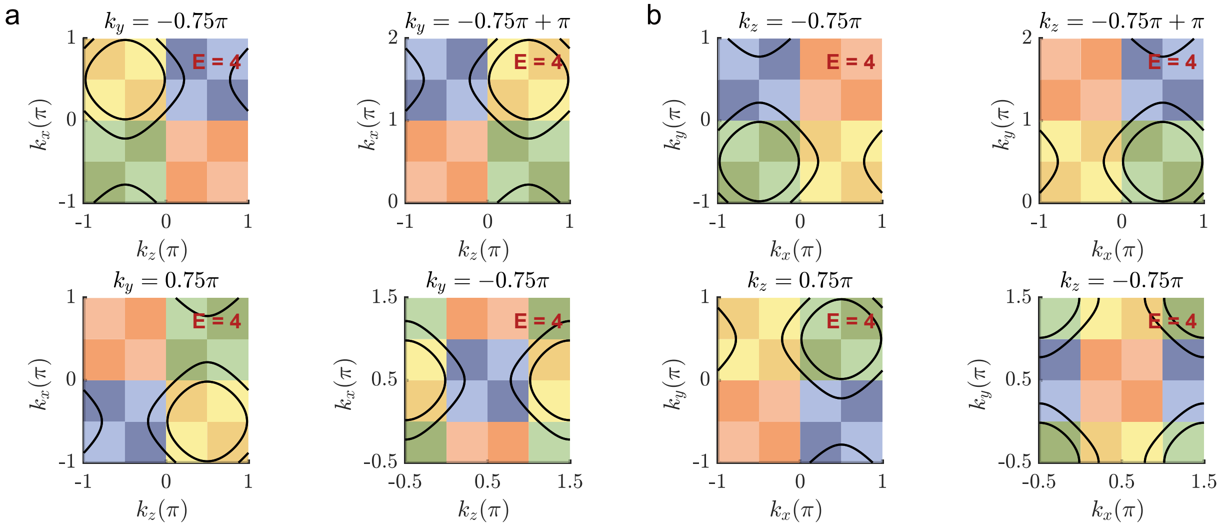

Supplementary Figure S4. Iso-energy contours at E=4 on the remaining $z - x$ plane and $x - y$ plane.

We validate the aforementioned symmetric operators by visualizing the iso-energy contours, specifically exemplifying the $y - z$ plane. The distinctive characteristic of the **k**-NS reflection $\mathcal{M}_y$ becomes apparent at fixed values of $k_x = -0.75\pi$ and $k_x = -0.75\pi + \pi$, the constraint imposed by which in Eq. (4) manifests as follows,

$$\mathcal{M}_y H(k_x, k_y, k_z)\mathcal{M}_y^\dagger = H(\pi + k_x, -k_y, \pi + k_z). \tag{12}$$

Then we derive $H(\pi+k_x, -k_y, \pi+k_z)\mathcal{M}_y |\psi(\mathbf{k})\rangle = E(\mathbf{k})\mathcal{M}_y |\psi(\mathbf{k})\rangle$,which indicates that $\mathcal{M}_y |\psi(\mathbf{k})\rangle$ is an eigenstate of $H(\pi+k_x, -k_y, \pi+k_z)$ and its eigenvalue $E(\mathbf{k})$ is identical to that of the eigenstate $|\psi(\mathbf{k})\rangle$ of $H(\mathbf{k})$. Therefore, the energy at (k_x, k_y, k_z) is equivalent to that at $(\pi + k_x, -k_y, \pi + k_z)$.

As illustrated in Figs. S3a and b, upon shifting (k_x, k_z) to $(k_x+\pi, k_z+\pi)$, the iso-energy contours of the two exhibit a simple mirror symmetry about $k_y = 0$. Given the conventional inversion operation $\mathcal{I}$, Figs. S3a and c exemplify the energy equivalence at (k_x, k_y, k_z) and $(-k_x, -k_y, -k_z)$. Figure S3d depicts the impact of $\mathbf{k}$-NS inversion $\mathcal{P}_{yz}$, under which the iso-energy contours respect inversion-symmetric about $(k_y, k_z) = (\pi/2, \pi/2)$. The iso-energy contours on the remaining $z-x$ plane and $x-y$ plane manifest analogous characteristics (Fig. S4).

Supplementary Note 3. Topological invariants calculated by nested Wilson loops

The corner states observed in the circuit of the $\mathbb{RP}^3$ HOTI are induced by the bulk octupole moment. For the system in a trivial state, the topological invariant stands at 0, contrasting with 0.5 for the non-trivial phase. Note that at the resonance frequency $\omega_0 = 1/\sqrt{L_1C_1}$, the diagonal elements of the circuit Laplacian $\tilde{J}_\lambda(\omega, \mathbf{k})$ vanish and take exactly the form of the TB Hamiltonian. Hence, we employ $\tilde{J}_\lambda(\omega_0, \mathbf{k})$to calculate the nested Wilson loops, which comprises three primary steps.

Given that our $\mathbb{RP}^3$ HOTI model has eight bands, and the octupole occurs at half-filling, the first round of the nested Wilson loops is calculated by integrating the occupied bands along k_x direction, which yields a $N_{occ} \times N_{occ}$ matrix, defined as

$$\mathcal{W}_{x,\mathbf{k}} = F_{\mathbf{k}} F_{\mathbf{k}+\Delta_{k_x}} \cdots F_{\mathbf{k}+(N_x-2)\Delta_{k_x}} F_{\mathbf{k}+(N_x-1)\Delta_{k_x}}. \tag{13}$$

Here, $[F_{\mathbf{k}}]^{mn} = \langle u^m_{\mathbf{k}} | u^n_{\mathbf{k}+\Delta_{k_x}} \rangle$ $(m, n = 1, 2, \cdots, N_{occ})$ is the Wilson line element, where $|u^n_{\mathbf{k}+\Delta_{k_x}}\rangle$ are the eigenstates of $\tilde{J}_\lambda(\omega_0, \mathbf{k})$, N_{occ} is the number of the occupied energy bands and in this case is 4, $\Delta_{k_x} = (2\pi/N_x, 0, 0)$ and N_x is the number of nodes in the x direction. Notably, $[F_{\mathbf{k}+(N_x-1)\Delta_{k_x}}]^{mn} = \langle u^m_{\mathbf{k}+(N_x-1)\Delta_{k_x}} | u^n_{\mathbf{k}} \rangle$, which is essential to form the entire loop. The calculated results can be formulated as $W_{x,\mathbf{k}} = e^{iH_{\text{surface}}(\mathbf{k})}$. The Hamiltonian $H_{\text{surface}}(\mathbf{k})$ possesses the same topological properties as the two-dimensional $y-z$ surface of the three-dimensional circuit.To characterize the boundary topology, we diagonalize this Wilson loop,

$$\mathcal{W}_{x,\mathbf{k}} |v^j_{x,\mathbf{k}}\rangle = e^{i2\pi\nu^j_x(k_y,k_z)} |v^j_{x,\mathbf{k}}\rangle. \tag{14}$$

For the eigenstates of the Wilson loop $|v^j_{x,\mathbf{k}}\rangle$, the subscript x denotes the direction of the Wilson loop, $j = 1, 2, \cdots, N_{occ}$, and $\mathbf{k}$ specifies its starting point. The phases $\nu^j_x(k_y, k_z)$ in Eq. (14) are the eigenvalues of the first round of nested Wilson loops, which depend on k_y and k_z. After the first round of the nested Wilson loops, we obtain two doubly degenerate Wannier bands, each

characterized by eigenvalues of opposite signs (Fig. S5a), gapped across the entire 2-dimensional BZ $(k_y, k_z) \in [0, 2\pi) \times [0, 2\pi)$. In this condition, we can define two Wannier bands

$$\begin{aligned} \nu_x^- &= \left\{\nu_x^j(k_y, k_z),\ \text{s.t. } \nu_x^j(k_y, k_z) \text{ is below the Wannier gap}\right\}, \\ \nu_x^+ &= \left\{\nu_x^j(k_y, k_z),\ \text{s.t. } \nu_x^j(k_y, k_z) \text{ is above the Wannier gap}\right\}, \end{aligned} \tag{15}$$

and choose the sector ν_x^+ to characterize its topology by the second round of the nested Wilson loops.

In the second round of the nested Wilson loops, we first construct the Wannier states $|w_{x,\mathbf{k}}^{+,j}\rangle = \sum_{n=1}^{N_{\rm occ}} |u_{\mathbf{k}}^n\rangle\, [v_{x,\mathbf{k}}^{+,j}]^n$ as a linear superposition of the eigenstates of the Wannier sector ν_x^+, and then use these states to calculate the integration along the k_y direction,

$$[\widetilde{\mathcal{W}}_{y,\mathbf{k}}^{+_x}]^{j,j'} = \langle w_{x,\mathbf{k}}^{+_x,j}|w_{x,\mathbf{k}+\Delta_{k_y}}^{+_x,r}\rangle \langle w_{x,\mathbf{k}+\Delta_{k_y}}^{+_x,r}|\cdots|w_{x,\mathbf{k}+(N_y-1)\Delta_{k_y}}^{+_x,s}\rangle \langle w_{x,\mathbf{k}+(N_y-1)\Delta_{k_y}}^{+_x,s}|w_{x,\mathbf{k}}^{+_x,j'}\rangle, \tag{16}$$

where $\Delta_{k_y} = (0, 2\pi/N_y, 0)$.The results of the second Wilson loop have an associated Hamiltonian $H_{\rm hinge}(\mathbf{k})$ and can be formulated as,

$$\widetilde{\mathcal{W}}_{y,\mathbf{k}}^{+_x} = e^{iH_{\rm hinge}(\mathbf{k})}. \tag{17}$$

The Hamiltonian $H_{\rm hinge}(\mathbf{k})$ possesses the same topological properties as those of the one-dimensional edges on the two-dimensional $y - z$ surface of the three-dimensional circuit. To characterize the topology of the one-dimensional boundary, we diagonalize this Wilson loop

$$\widetilde{\mathcal{W}}_{y,\mathbf{k}}^{+_x}\, |\eta_{y,\mathbf{k}}^{+_x,\pm}\rangle = e^{i2\pi\eta_y^{\pm}(k_z)}\, |\eta_{y,\mathbf{k}}^{+_x,\pm}\rangle. \tag{18}$$

The outcome of the second round of integration yields two non-degenerate bands with eigenvalues that are negatives of each other (Fig. S5b), which indicates $H_{\rm hinge}(\mathbf{k})$ is gapped. This topology is hosted by a 1D TI, so we can further define the two Wannier sectors $|w_{y,\mathbf{k}}^{+_x,+_y}\rangle = \sum_{n=1}^{N_{\rm occ}} |u_{x,\mathbf{k}}^{\pm,n}\rangle\, [\eta_{y,\mathbf{k}}^{+_x,\pm}]^n$, and use the η_y^+ to calculate the third round of the nested Wilson loop along k_z,

$$\widetilde{\mathcal{W}}_{z,\mathbf{k}}^{+_x,+_y} = \langle w_{y,\mathbf{k}}^{+_x,+_y}|w_{y,\mathbf{k}+\Delta_{k_z}}^{+_x,+_y}\rangle \langle w_{y,\mathbf{k}+\Delta_{k_z}}^{+_x,+_y}|\cdots|w_{y,\mathbf{k}+(N_z-1)\Delta_{k_z}}^{+_x,+_y}\rangle \langle w_{y,\mathbf{k}+(N_z-1)\Delta_{k_z}}^{+_x,+_y}|w_{y,\mathbf{k}}^{+_x,+_y}\rangle. \tag{19}$$

The results of the third round are given by the following equation,

$$p_z^{+_x,+_y} = |-\frac{i}{2\pi}\log(\widetilde{\mathcal{W}}_{z,\mathbf{k}}^{+_x,+_y})| = \begin{cases} 0 \quad, & \text{if } |\eta| > 1 \\ 1/2 \ \ , & \text{if } |\eta| < 1 \end{cases}. \tag{20}$$

Figure S5c illustrates the calculation results for the topological invariants, clearly demonstrating that for $|\eta| > 1$, the topological invariant is zero, corresponding to a topological trivial state; conversely, when $|\eta| < 1$, the topological invariant is 0.5, indicating a topological non-trivial state.

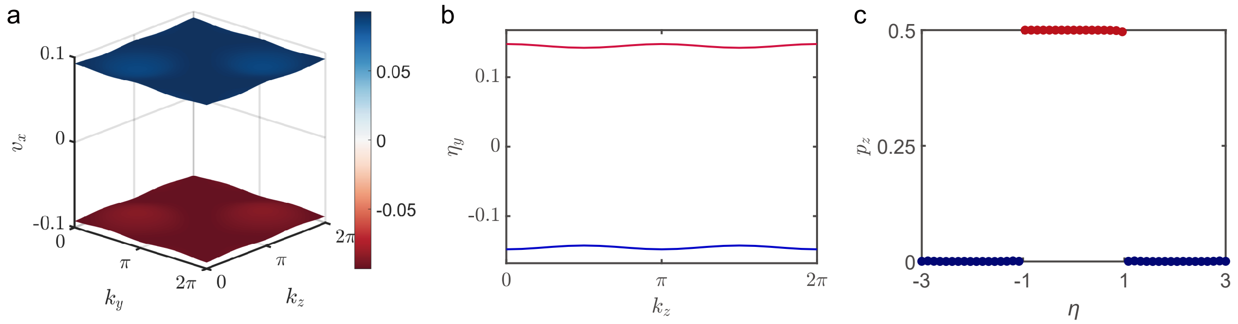

Supplementary Figure S5. Procedure to calculate the topological invariant of the bulk circuit with $\eta = 1/3.3$ through the nested Wilson loops. **a** Phase of the eigenvalue $v_x^{\pm,j}(k_y, k_z)$ derived from the first round nested Wilson loops along k_x. **b** Phase of theeigenvalue $\eta_y^{\pm,j}(k_z)$ derived from the second round nested Wilson loops along k_y. **c** Polarization $p_z^{\pm}$ with different η obtained from the phase of the third nested Wilson loop along k_z.

Supplementary Note 4. Topological invariants with symmetry constraint

Under the constraint of **k**-NS $M_x\,(k_x, k_y, k_z) \rightarrow (-k_x, k_y+\pi, k_z+\pi)$, the Bloch Hamiltonian $H(\mathbf{k})$ satisfies,

$$\mathcal{M}_x H(\mathbf{k}) \mathcal{M}_x^{\dagger} = H\left(M_x \mathbf{k}\right). \tag{21}$$

which operates on the eigenstates $|u_{\mathbf{k}}^n\rangle$ as

$$\mathcal{M}_x H(\mathbf{k}) |u_{\mathbf{k}}^n\rangle = H(M_x\mathbf{k})\mathcal{M}_x|u_{\mathbf{k}}^n\rangle = \epsilon_{n,\mathbf{k}}\mathcal{M}_x|u_{\mathbf{k}}^n\rangle. \tag{22}$$

We can expand the state $\mathcal{M}_x|u_{\mathrm{k}}^n\rangle$ with the eigenstates $|u_{M_x\mathrm{k}}^m\rangle$ at $M_x\mathbf{k}$,

$$\mathcal{M}_x|u_{\mathbf{k}}^n\rangle = |u_{M_x\mathbf{k}}^m\rangle\langle u_{M_x\mathbf{k}}^m|\mathcal{M}_x|u_{\mathbf{k}}^n\rangle \equiv |u_{M_x\mathbf{k}}^m\rangle B_{\mathcal{M}_x,\mathbf{k}}^{mn}, \tag{23}$$

where the summation over the indices m runs over the occupied bands. We can then define the screw matrix

$$B_{\mathcal{M}_x,\mathbf{k}}^{mn} \equiv \langle u_{M_x\mathbf{k}}^m|\mathcal{M}_x|u_{\mathbf{k}}^n\rangle. \tag{24}$$

Hence, the **k**-NS M_x transforms a discrete Wilson line element as

$$\langle u_{\mathbf{k}+\Delta_{\mathbf{k}}}^m \mid u_{\mathbf{k}}^n\rangle = B_{\mathcal{M}_x,\mathbf{k}+\Delta_{\mathbf{k}}}^{\dagger ml}\langle u_{M_x\mathbf{k}+\Delta_{\mathbf{k}}}^l \mid u_{M_x\mathbf{k}}^r\rangle B_{\mathcal{M}_x,\mathbf{k}}^{rn}, \tag{25}$$

where the indices r and l should be summed up. Furthermore, we can derive the transformation of the Wilson loop along x direction

$$\begin{aligned} B_{\mathcal{M}_x,\mathbf{k}}\mathcal{W}_{x,\mathbf{k}}B^\dagger_{\mathcal{M}_x,\mathbf{k}} &= \mathcal{W}_{-x,M_x\mathbf{k}}, \\ B_{\mathcal{M}_y,\mathbf{k}}\mathcal{W}_{x,\mathbf{k}}B^\dagger_{\mathcal{M}_y,\mathbf{k}} &= \mathcal{W}_{x,M_y\mathbf{k}}, \\ B_{\mathcal{M}_z,\mathbf{k}}\mathcal{W}_{x,\mathbf{k}}B^\dagger_{\mathcal{M}_z,\mathbf{k}} &= \mathcal{W}_{x,M_z\mathbf{k}}, \end{aligned} \tag{26}$$

implying that under the constraint of $\mathcal{M}_x$, the Wilson loop at $\mathbf{k}$ is equivalent to the Hermitian conjugate of the Wilson loop at $M_x\mathbf{k}$,

$$\left\{e^{i2\pi\nu_x^j(k_y,k_z)}\right\} \overset{M_x}{=} \left\{e^{-i2\pi\nu_x^j(k_y+\pi,k_z+\pi)}\right\}. \tag{27}$$

Hence, the Wannier centers obey

$$\{v_x^j(k_y,k_z)\} \overset{M_x}{=} \{-v_x^j(k_y+\pi,k_z+\pi)\}\ mod\ 1. \tag{28}$$

A similar approach is valid for a nested Wilson loop, and the screw matrix is defined by the operator

$$A^{ij}_{\mathcal{M}_x,\mathbf{k}} \equiv \langle v^i_{M_x\mathbf{k}}|B_{\mathcal{M}_x,\mathbf{k}}|v^j_{\mathbf{k}}\rangle. \tag{29}$$

The transformation of the nested Wilson loop along y direction denotes

$$\begin{aligned} A_{\mathcal{M}_x,\mathbf{k}}\widetilde{\mathcal{W}}^{+x}_{y,\mathbf{k}}A^\dagger_{\mathcal{M}_x,\mathbf{k}} &= \widetilde{\mathcal{W}}^{+x}_{y,M_x\mathbf{k}}, \\ A_{\mathcal{M}_y,\mathbf{k}}\widetilde{\mathcal{W}}^{+x}_{y,\mathbf{k}}A^\dagger_{\mathcal{M}_y,\mathbf{k}} &= \widetilde{\mathcal{W}}^{+x}_{-y,M_y\mathbf{k}}, \\ A_{\mathcal{M}_z,\mathbf{k}}\widetilde{\mathcal{W}}^{+x}_{y,\mathbf{k}}A^\dagger_{\mathcal{M}_z,\mathbf{k}} &= \widetilde{\mathcal{W}}^{+x}_{y,M_z\mathbf{k}}. \end{aligned} \tag{30}$$

implying that under the constraint of $\mathcal{M}_y$, the nested Wannier centers are subject to

$$\{\eta_y^{+x}(k_z)\} \overset{M_y}{=} \{-\eta_y^{+x}(k_z+\pi)\}\ mod\ 1. \tag{31}$$

For the third round of nested Wilson loop along z direction, the screw matrix is defined as

$$O^{st}_{\mathcal{M}_x,\mathbf{k}} \equiv \langle \eta^s_{M_x\mathbf{k}}|A_{\mathcal{M}_z,\mathbf{k}}|\eta^t_{\mathbf{k}}\rangle, \tag{32}$$

and the corresponding transformations are

$$\begin{aligned} O_{\mathcal{M}_x,\mathbf{k}}\widetilde{\mathcal{W}}^{+x,+y}_{z,\mathbf{k}}O^\dagger_{\mathcal{M}_x,\mathbf{k}} &= \widetilde{\mathcal{W}}^{+x,+y}_{z,M_x\mathbf{k}}, \\ O_{\mathcal{M}_y,\mathbf{k}}\widetilde{\mathcal{W}}^{+x,+y}_{z,\mathbf{k}}O^\dagger_{\mathcal{M}_y,\mathbf{k}} &= \widetilde{\mathcal{W}}^{+x,+y}_{z,M_y\mathbf{k}}, \\ O_{\mathcal{M}_z,\mathbf{k}}\widetilde{\mathcal{W}}^{+x,+y}_{z,\mathbf{k}}O^\dagger_{\mathcal{M}_z,\mathbf{k}} &= \widetilde{\mathcal{W}}^{+x,+y}_{-z,M_z\mathbf{k}}, \end{aligned} \tag{33}$$

implying that under the constraint of $\mathcal{M}_y$, the doubly nested Wannier centers are subject to

$$\{\eta_z^{+x,+y}\} \overset{M_z}{=} \{-\eta_z^{+x,+y}\} \bmod 1, \tag{34}$$

which quantizes $\eta_z^{\pm x,\pm y}$ to 0 or 1/2. The symmetry operator $\mathcal{M}_x$, $\mathcal{M}_y$, and $\mathcal{M}_z$ give the quantization

$$\begin{aligned}\left(p_x^{+y,+z}, p_y^{+x,+z}, p_z^{+x,+y}\right) = (0,0,0)\,, \left(\frac{1}{2},0,0\right), \left(0,\frac{1}{2},0\right), \left(0,0,\frac{1}{2}\right), \\ \left(\frac{1}{2},\frac{1}{2},0\right), \left(\frac{1}{2},0,\frac{1}{2}\right), \left(0,\frac{1}{2},\frac{1}{2}\right), \left(\frac{1}{2},\frac{1}{2},\frac{1}{2}\right)\end{aligned} \tag{35}$$

and leads to a quantized octupole moment

$$Q_{xyz} = \sum_{\pm} p_x^{\pm y,\pm z} p_y^{\pm x,\pm z} p_z^{\pm x,\pm y} = 0 \text{ or } \frac{1}{2}. \tag{36}$$

Supplementary Note 5. Parameter tuning in semi-open circuit systems.

In the main text, we investigated the effect of tuning the hopping strengths in the periodic direction on the boundary states under semi-open boundary conditions (Figs. 4a-c in the main text). Similarly, we map the model from the electronic system to the circuit system ,and conduct a study of the parameter space and band structure analysis in the semi-open circuit system as well. We found that, although dispersion exists, the band structure and its trend of variation in the circuit system are consistent with those in the electronic system within a certain frequency range (around the resonance frequency $\omega_0 = 2.77$MHz). As shown in Figs. S6d-e (the upper panels correspond to the electronic system, while the lower panels correspond to the circuit system), when two boundaries are periodic (in the x and y directions) and one direction is open (z direction), surface states and bulk states coexist. By varying the hopping strength in the x and y directions simultaneously, the band gap closes and a phase transition occurs when crossing the hinge $|\eta_x| = |\eta_y| = 1$ in the parameter space.

In addition to analyzing the band structure of the circuit system under semi-open conditions, we use end-to-end connections to simulate PBCs and draw the density of states of edge states in different dimensions for circuit systems with $20 \times 20 \times 20$ nodes. Figure S7a shows the distribution of corner states in the full open system; by connecting the $x - y$ planes, we create a $\mathbb{RP}^3$ HOTI system with OBCs in the x and y directions and PBCs in the z direction, where hinge states are present along the z direction (Fig. S7b); similarly, by connecting the $x - z$ planes and $y - z$ planes, respectively, we establish a system with OBCs in the z direction and PBCs in the x and y directions, resulting in surface states appearing on the $x - y$ planes (Fig. S7c).

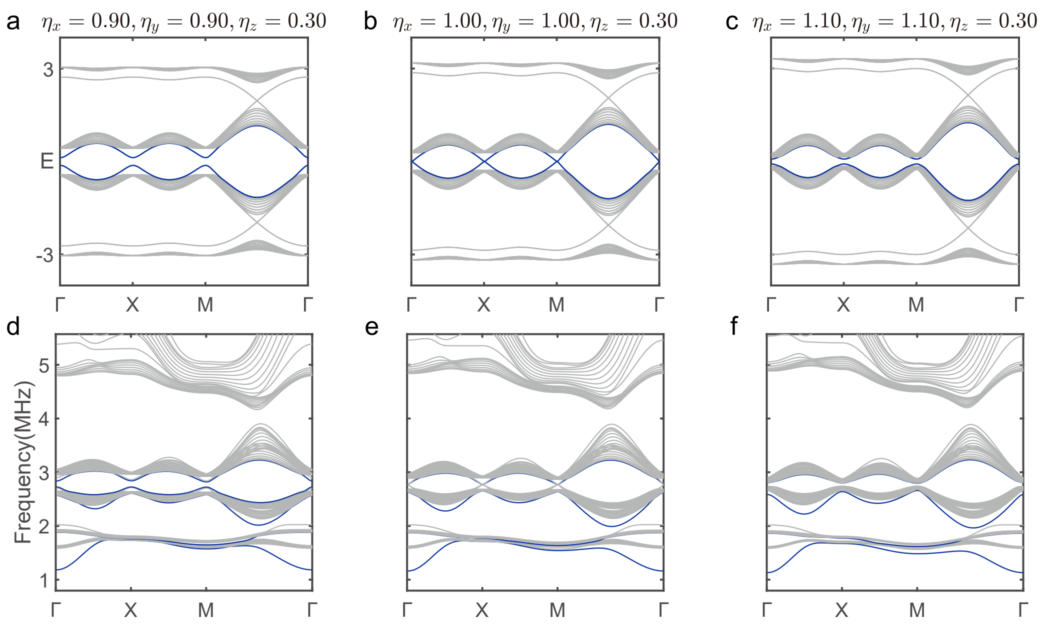

Supplementary Figure S6. In the $\mathbb{RP}^3$ HOTI system with OBCs in the x direction and PBCs in the y and z directions, the comparison of phase transition results between the electronic system (upper panels) and the circuit system (lower panels) under the same parameters. **a**, **d** Topological non-trivial phase. **b**, **e** The phase-transition point on the hinges $|\eta_y| = |\eta_z| = 1$. **c**, **f** Topological trivial phase.

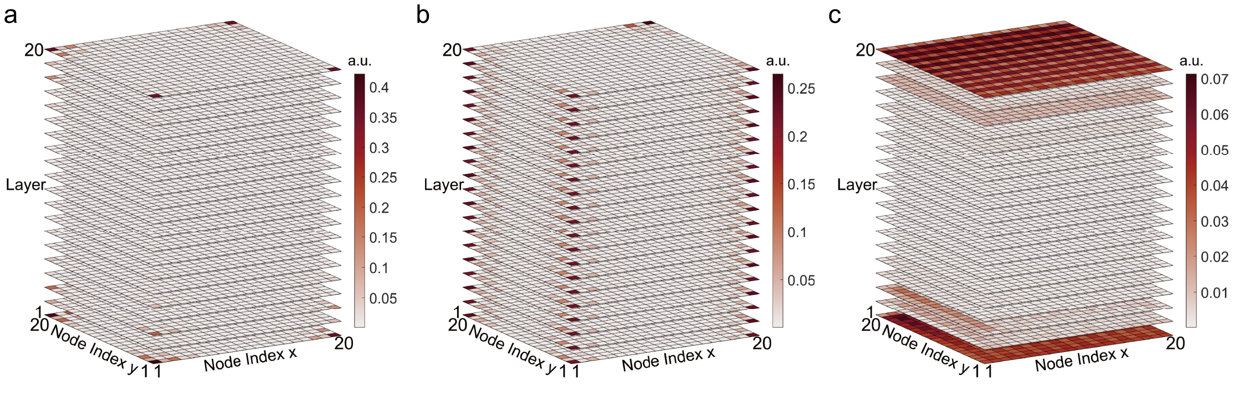

Supplementary Figure S7. Density of states(DOS) in 3D $\mathbb{RP}^3$ topological circuit systems with $N_x = N_y = N_z = 20$. **a** Corner states of the full-open system. **b** Hinge states along the z direction of the semi-open system (OBCs in the x and y directions, PBCs in the z direction). **c** Surface states on the $x - y$ planes of the semi-open system (OBCs in the z directions, PBCs in the x and y directions).

Supplementary Note 6. Topology of the finite circuit with a complete period

To realize the octupole insulating phase located at the corner of the bulk circuit, it is imperative that two specific criteria should be fulfilled. First, the boundary should preserve the symmetries that protect the topological characteristics of the system. Subsequently, the boundary termination should not intersect the unit cell, thereby maintaining the structural integrity of the lattice. To satisfy the first requirement, we ensure the vanishing of the diagonal elements of $\tilde{J}_\lambda(\omega, \mathbf{k})$, thus preserving the symmetries of the topological circuit. Regarding the second requirement, it is achieved by designing the layout so that the corner terminates at a unit cell boundary, thus avoiding any disruption to the integrity of the unit cell. Considering that the circuit framework presented in the main text consists of $2.5 \times 2.5 \times 2.5$ unit cells, which means that only one corner meets the criteria for hosting octupole corner states, we herein introduce a circuit model with $3 \times 3 \times 3$ unit cells (Fig. S8a) where all eight corners satisfy the condition for the emergence of such states.

Figure S8b and c depict the eigenvalues of the circuit Laplacian $J(\omega)$ and the dynamic D matrix of the circuit Hamiltonian, respectively. When the eigenvalue $j_n(\omega)$ equals zero, eight isolated modes can be observed (Fig. S8b), corresponding to the corner states in the band gap as shown in Fig. S8c. It is noteworthy that these eight corner states are not in complete degeneracy but rather form two sets, located at two closed but different frequencies. This phenomenon stems from the distinctive eigenvalue transitions of $\mathbb{RP}^3$ HOTI under OBCs, which differ from those under PBCs. Returning to the initial tight-binding model with intra-cell coupling v and inter-cell coupling w, for the semi-infinite scenario, where some directions have periodic boundaries while the other terminates, the boundary state emerges only in the terminated direction, appearing near $E = 0$. Due to the chiral symmetry of the Hamiltonian, for every mode at $E = +E_0$, there is a corresponding mode at $E = -E_0$, ensuring that the isolated boundary state appears strictly at $E = 0$ and transitions strictly at $v = 1$. When one considers the finite-size system, with OBCs in every direction, a boundary state appears in each direction (i.e., each corner), and each has a certain decay length, leading to minor couplings between them. Such interactions cause a deviation in the transition point away from $v = 1$, potentially down to $v = 0$. Despite increasing the size of the system, coupling effects remain, propelling the boundary states to appear on both sides of $E = 0$ consistent with chiral symmetry, until they degenerate strictly at $v = 0$, which is a fully dimerized state.

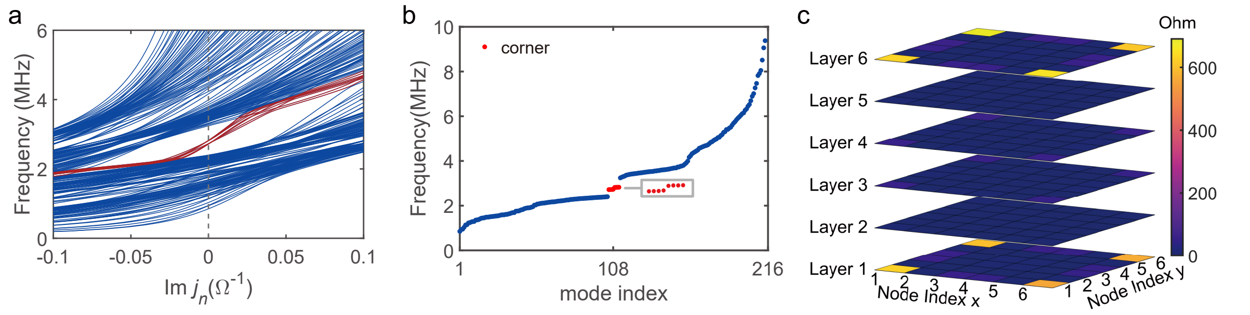

Supplementary Figure S8. The theoretical model and topological properties of the finite circuit with $3 \times 3 \times 3$ unit cells. **a** Schematic of the finite circuit. **b** Eigenvalue spectrum of $J(\omega)$. The zero-energy eigenvalue at resonant frequency 2.77 MHz corresponds to the corner states (red dots) in **c**. **c** Eigenfrequencies of the finite circuit characterized by D matrix.

Supplementary Note 7. Details for the experimental circuit

For an infinite $\mathbb{RP}^3$ topological circuit composed of the unit cell in Fig. 2a, the capacitance matrix C and inductance matrix W are given as,

$$C = \begin{bmatrix} -(3C_1+C_2) & 0 & C_1 & C_1+C_2e^{iky} & C_1 & 0 & 0 & 0 \\ 0 & -(2C_1+2C_2) & C_2e^{-iky} & C_1+C_2e^{ikx} & 0 & C_1 & 0 & 0 \\ C_1 & C_2e^{iky} & -(2C_1+2C_2+C_{1g}) & 0 & 0 & 0 & C_1+C_2e^{ikz} & 0 \\ C_1+C_2e^{-iky} & C_1+C_2e^{-ikx} & 0 & -(3C_1+3C_2) & 0 & 0 & 0 & C_1+C_2e^{ikz} \\ C_1 & 0 & 0 & 0 & -(C_1+C_2) & 0 & 0 & C_2e^{iky} \\ 0 & C_1 & 0 & 0 & 0 & -(2C_1+2C_2) & C_1+C_2e^{-iky} & C_2e^{ikx} \\ 0 & 0 & C_1+C_2e^{-ikz} & 0 & 0 & C_1+C_2e^{iky} & -(2C_1+2C_2+C_{2g}) & 0 \\ 0 & 0 & 0 & C_1+C_2e^{-ikz} & C_2e^{-iky} & C_2e^{-ikx} & 0 & -(C_1+3C_2) \end{bmatrix}, \tag{37}$$

$$W = \begin{bmatrix} -(2/L_2+1/L_{1g}) & 0 & 1/L_2e^{-ikx} & 0 & 1/L_2e^{ikz} & 0 & 0 & 0 \\ 0 & -(1/L_1+1/L_2+1/L_{2g}) & 1/L_1 & 0 & 0 & 1/L_2e^{ikz} & 0 & 0 \\ 1/L_2e^{ikx} & 1/L_1 & -(1/L_1+1/L_2) & 0 & 0 & 0 & 0 & 0 \\ 0 & 0 & 0 & -(1/L_{2g}) & 0 & 0 & 0 & 0 \\ 1/L_2e^{-ikz} & 0 & 0 & 0 & -(2/L_1+2/L_2+1/L_{3g}) & 0 & 1/L_1+1/L_2e^{-ikx} & 1/L_1 \\ 0 & 1/L_2e^{-ikz} & 0 & 0 & 0 & -(1/L_1+1/L_2+1/L_{1g}) & 0 & 1/L_1 \\ 0 & 0 & 0 & 0 & 1/L_1+1/L_2e^{ikx} & 0 & -(1/L_1+1/L_2) & 0 \\ 0 & 0 & 0 & 0 & 1/L_1 & 1/L_1 & 0 & -(1/L_1+1/L_2+1/L_{2g}) \end{bmatrix}. \tag{38}$$

According to Kirchhoff's circuit laws, any electrical circuit network can be fully described by the circuit Laplacian matrix $J(\omega)$,

$$J(\omega) = i\omega C + \frac{1}{\omega}W, \tag{39}$$

where C and W are the capacitance matrix and inverse inductance matrix of the circuit system,

respectively. The diagonal and off-diagonal components represent the self-admittance of a certain node and mutual admittance between two nodes, respectively. $J(\omega)$ is purely imaginary when there are only capacitors and inductors in the $\mathbb{RP}^3$ circuit. And the relationship between the current and voltage in the circuit is expressed as:

$$J(\omega)V = I, \tag{40}$$

which holds a similar form to the eigenvalue equation $H\varphi = E\varphi$. Below we provide a detailed derivation to demonstrate that the circuit Laplacian $J(\omega)$ at the resonant frequency ω_0 corresponds exactly to the Hamiltonian in Eq.(4) of the main text.

At the resonant frequency ω_0, we have $i\omega_0 C_1 + 1/i\omega_0 L_1 = i\omega_0 C_2 + 1/i\omega_0 L_2 = 0$. Replacing the inductances L_1 and L_2 with C_1 and C_2 in the expression, $J(\omega_0)$ is given as

$$\begin{aligned} J(\omega_0) &= i\omega_0 C + \frac{1}{i\omega_0}W \\ &= i\omega_0 C_1 \begin{bmatrix} 0 & 0 & 1-\lambda e^{-ikx} & 1+\lambda e^{iky} & 1-\lambda e^{ikz} & 0 & 0 & 0 \\ 0 & 0 & -1+\lambda e^{-iky} & 1+\lambda e^{ikx} & 0 & 1-\lambda e^{ikz} & 0 & 0 \\ 1-\lambda e^{ikx} & -1+\lambda e^{iky} & 0 & 0 & 0 & 0 & 1+\lambda e^{ikz} & 0 \\ 1+\lambda e^{-iky} & 1+\lambda e^{-ikx} & 0 & 0 & 0 & 0 & 0 & 1+\lambda e^{ikz} \\ 1-\lambda e^{-ikz} & 0 & 0 & 0 & 0 & 0 & -1-\lambda e^{-ikx} & -1+\lambda e^{iky} \\ 0 & 1-\lambda e^{-ikz} & 0 & 0 & 0 & 0 & 1+\lambda e^{-iky} & -1+\lambda e^{ikx} \\ 0 & 0 & 1+\lambda e^{-ikz} & 0 & -1-\lambda e^{ikx} & 1+\lambda e^{iky} & 0 & 0 \\ 0 & 0 & 0 & 1+\lambda e^{-ikz} & -1+\lambda e^{-iky} & -1+\lambda e^{-ikx} & 0 & 0 \end{bmatrix}, \end{aligned} \tag{41}$$

where $\lambda = C_2/C_1$.The Hamiltonian of the electronic system in Eq. (4) can be represented more intuitively in matrix form:

$$H = \begin{bmatrix} 0 & 0 & 1-\lambda e^{-ikx} & 1+\lambda e^{iky} & 1-\lambda e^{ikz} & 0 & 0 & 0 \\ 0 & 0 & -1+\lambda e^{-iky} & 1+\lambda e^{ikx} & 0 & 1-\lambda e^{ikz} & 0 & 0 \\ 1-\lambda e^{ikx} & -1+\lambda e^{iky} & 0 & 0 & 0 & 0 & 1+\lambda e^{ikz} & 0 \\ 1+\lambda e^{-iky} & 1+\lambda e^{-ikx} & 0 & 0 & 0 & 0 & 0 & 1+\lambda e^{ikz} \\ 1-\lambda e^{-ikz} & 0 & 0 & 0 & 0 & 0 & -1-\lambda e^{-ikx} & -1+\lambda e^{iky} \\ 0 & 1-\lambda e^{-ikz} & 0 & 0 & 0 & 0 & 1+\lambda e^{-iky} & -1+\lambda e^{ikx} \\ 0 & 0 & 1+\lambda e^{-ikz} & 0 & -1-\lambda e^{ikx} & 1+\lambda e^{iky} & 0 & 0 \\ 0 & 0 & 0 & 1+\lambda e^{-ikz} & -1+\lambda e^{-iky} & -1+\lambda e^{-ikx} & 0 & 0 \end{bmatrix}. \tag{42}$$

By comparing Eq. (41) and Eq. (42, it can be seen that the circuit Laplacian $J(\omega)$ at the resonant frequency ω_0 differs from the Hamiltonian of the electronic system only by a constant factor. Thus, the effective model of the circuit corresponds to Eq. (4).

Figure S9a gives the detailed 5 layers for the finite circuit model constructed in the main text, and the experimental measurement platform is shown in Fig. S9b. The following section presents the grounding terms for the unit cell circuit under PBCs, as well as the grounded terms for the finite circuit model, which consists of five layers with 125 nodes in total.

Supplementary Table 1. Grounded terms for the unit cell circuit ($\lambda = 3.3$)

Node index	Grounded term	Node index	Grounded term
1	$L_{1g} = L_1/(3+3\lambda)$	5	$L_{3g} = L_1/(3\lambda - 1)$
2	$L_{2g} = L_1/(1+\lambda)$	6	$L_{2g} = L_1/(1+\lambda)$
3	$C_{1g} = C_2 - 3C_1$	7	$C_{2g} = C_1 + C_2$
4	$L_{2g} = L_1/(1+\lambda)$	8	$L_{2g} = L_1/(1+\lambda)$

The theoretical values of grounded terms

Node index	Grounded term	Node index	Grounded term
1	$L_{1g} = 0.256\mu H$	5	$L_{3g} = 0.371\mu H$
2	$L_{2g} = 0.767\mu H$	6	$L_{2g} = 0.767\mu H$
3	$C_{1g} = 0.3nF$	7	$C_{2g} = 4.3nF$
4	$L_{2g} = 0.767\mu H$	8	$L_{2g} = 0.767\mu H$

Supplementary Table 2. Grounded terms for the finite circuit at layer 1

	1	2	3	4	5
5	$L_{\text{corner\_3}} = L_2$	$L_{x01} = L_2/2$	$L_{x01} = L_2/2$	$L_{x01} = L_2/2$	$L_{\text{corner\_4}} = L_1/(2\lambda+1)$
4	$L_{y00} = L_1/(\lambda - 1)$	$L_{xoy0\_4} = L_1$	$C_{xoy0\_3} = C_1$	$L_{xoy0\_4} = L_1$	$C_{y01\_2} = C_1$<br>$L_{y01\_2} = L_1$
3	$L_{y00} = L_1/(\lambda - 1)$	$L_{xoy0\_2} = L_1/(2\lambda+1)$	$L_{xoy0\_1} = L_1/(2\lambda-1)$	$L_{xoy0\_2} = L_1/(2\lambda+1)$	$L_{y01\_1} = L_2/2$
2	$L_{y00} = L_1/(\lambda - 1)$	$L_{xoy0\_4} = L_1$	$C_{xoy0\_3} = C_1$	$L_{xoy0\_4} = L_1$	$C_{y01\_2} = C_1$<br>$L_{y01\_2} = L_1$
1	$C_{\text{corner\_1}} = C_1$	$L_{x00\_2} = L_1/(1+\lambda)$	$L_{x00\_1} = L_1/(\lambda - 1)$	$L_{x00\_2} = L_1/(1+\lambda)$	$L_{\text{corner\_2}} = L_2$

The theoretical values of grounded terms

	1	2	3	4	5
5	$L_{\text{corner\_3}} = 1\mu H$	$L_{x01} = 0.5\mu H$	$L_{x01} = 0.5\mu H$	$L_{x01} = 0.5\mu H$	$L_{\text{corner\_4}} = 0.434\mu H$
4	$L_{y00} = 1.435\mu H$	$L_{xoy0\_4} = 3.3\mu H$	$C_{xoy0\_3} = 1nF$	$L_{xoy0\_4} = 1nF$	$C_{y01\_2} = 1nF$<br>$L_{y01\_2} = 3.3\mu H$
3	$L_{y00} = 1.435\mu H$	$L_{xoy0\_2} = 0.434\mu H$	$L_{xoy0\_1} = 0.589\mu H$	$L_{xoy0\_2} = 0.434\mu H$	$L_{y01\_1} = 0.5\mu H$
2	$L_{y00} = 1.435\mu H$	$L_{xoy0\_4} = 3.3\mu H$	$C_{xoy0\_3} = 1nF$	$L_{xoy0\_4} = 3.3\mu H$	$C_{y01\_2} = 1nF$<br>$L_{y01\_2} = 3.3\mu H$
1	$C_{\text{corner\_1}} = 1nF$	$L_{x00\_2} = 0.767\mu H$	$L_{x00\_1} = 1.435\mu H$	$L_{x00\_2} = 0.767\mu H$	$L_{\text{corner\_2}} = 1\mu H$

Supplementary Table 3. Grounded terms for the finite circuit at layer 2

	1	2	3	4	5
5	$L_{z10\_2} = L_1/(2\lambda+2)$	$L_{xoz1\_2} = L_1/(\lambda+2)$	$L_{xoz1\_1} = L_1/(3\lambda+2)$	$L_{xoz1\_2} = L_1/(\lambda+2)$	$L_{z11} = L_1/(3\lambda+1)$
4	$L_{yoz0\_2} = L_1/3$	$L_{2g} = L_1/(\lambda+1)$	$C_{1g} = C_2 - 3C_1$	$L_{2g} = L_1/(\lambda+1)$	$C_{yoz1\_2} = C_2 - 2C_1$
3	$L_{yoz0\_1} = L_1/(2\lambda+3)$	$L_{2g} = L_1/(\lambda+1)$	$L_{1g} = L_1/(3\lambda+3)$	$L_{2g} = L_1/(\lambda+1)$	$L_{yoz1\_1} = L_1/(3\lambda+2)$
2	$L_{yoz0\_2} = L_1/3$	$L_{2g} = L_1/(\lambda+1)$	$C_{1g} = C_2 - 3C_1$	$L_{2g} = L_1/(\lambda+1)$	$C_{yoz1\_2} = C_2 - 2C_1$
1	$L_{z00\_2} = L_1/(\lambda+3)$	$L_{xoz0\_2} = L_1$	$L_{xoz0\_1} = L_1/(2\lambda+3)$	$L_{xoz0\_2} = L_1$	$L_{z01\_2} = L_1/(2\lambda+2)$

The theoretical values of grounded terms

	1	2	3	4	5
5	$L_{z10\_2} = 0.384\mu H$	$L_{xoz1\_2} = 0.623\mu H$	$L_{xoz1\_1} = 0.277\mu H$	$L_{xoz1\_2} = 0.623\mu H$	$L_{z11} = 0.303\mu H$
4	$L_{yoz0\_2} = 1.1\mu H$	$L_{2g} = 0.767\mu H$	$C_{1g} = 0.3nF$	$L_{2g} = 0.767\mu H$	$C_{yoz1\_2} = 1.3nF$
3	$L_{yoz0\_1} = 0.344\mu H$	$L_{2g} = 0.767\mu H$	$L_{1g} = 0.256\mu H$	$L_{2g} = 0.767\mu H$	$L_{yoz1\_1} = 0.277\mu H$
2	$L_{yoz0\_2} = 1.1\mu H$	$L_{2g} = 0.767\mu H$	$C_{1g} = 0.3nF$	$L_{2g} = 0.767\mu H$	$C_{yoz1\_2} = 1.3nF$
1	$L_{z00\_2} = 0.524\mu H$	$L_{xoz0\_2} = 3.3\mu H$	$L_{xoz0\_1} = 0.344\mu H$	$L_{xoz0\_2} = 3.3\mu H$	$L_{z01\_2} = 0.384\mu H$

Supplementary Table 4. Grounded terms for the finite circuit at layer 3

	1	2	3	4	5
5	$L_{z10\_1} = L_1/(2\lambda+2)$	$L_{xoz1\_4} = L_2$	$L_{xoz1\_3} = L_2/3$	$L_{xoz1\_4} = L_2$	$L_{z11} = L_1/(3\lambda+1)$
4	$C_{yoz0\_4} = C_1$	$L_{2g} = L_1/(\lambda+1)$	$C_{2g} = C_1 + C_2$	$L_{2g} = L_1/(\lambda+1)$	$C_{yoz1\_4} = C_2$
3	$L_{yoz0\_3} = L_1/(2\lambda-1)$	$L_{2g} = L_1/(\lambda+1)$	$L_{3g} = L_1/(3\lambda-1)$	$L_{2g} = L_1/(\lambda+1)$	$L_{yoz1\_3} = L_2/3$
2	$C_{yoz0\_4} = C_1$	$L_{2g} = L_1/(\lambda+1)$	$C_{2g} = C_1 + C_2$	$L_{2g} = L_1/(\lambda+1)$	$C_{yoz1\_4} = C_2$
1	$L_{z00\_1} = L_1/(\lambda-1)$	$L_{xoz0\_2} = L_1$	$L_{xoz0\_3} = L_1/(2\lambda-1)$	$L_{xoz0\_2} = L_1$	$L_{z01\_1} = L_2/2$

The theoretical values of grounded terms

	1	2	3	4	5
5	$L_{z10\_1} = 0.384\mu H$	$L_{xoz1\_4} = 1\mu H$	$L_{xoz1\_3} = 0.333\mu H$	$L_{xoz1\_4} = 1\mu H$	$L_{z11} = 0.303\mu H$
4	$C_{yoz0\_4} = 1nF$	$L_{2g} = 0.767\mu H$	$C_{2g} = 4.3nF$	$L_{2g} = 0.767\mu H$	$C_{yoz1\_4} = 3.3nF$
3	$L_{yoz0\_3} = 0.589\mu H$	$L_{2g} = 0.767\mu H)$	$L_{3g} = 0.371\mu H$	$L_{2g} = 0.767\mu H$	$L_{yoz1\_3} = 0.333\mu H$
2	$C_{yoz0\_4} = 3.3nF$	$L_{2g} = 0.767\mu H$	$C_{2g} = 4.3nF$	$L_{2g} = 0.767\mu H$	$C_{yoz1\_4} = 3.3nF$
1	$L_{z00\_1} = 1.435\mu H$	$L_{xoz0\_2} = 3.3\mu H$	$L_{xoz0\_3} = 0.589\mu H$	$L_{xoz0\_2} 3.3\mu H$	$L_{z01\_1} = 0.5\mu H$

Supplementary Table 5. Grounded terms for the finite circuit at layer 4

	1	2	3	4	5
5	$L_{z10\_2} = L_1/(2\lambda+2)$	$L_{xoz1\_2} = L_1/(\lambda+2)$	$L_{xoz1\_1} = L_1/(3\lambda+2)$	$L_{xoz1\_2} = L_1/(\lambda+2)$	$L_{z11} = L_1/(3\lambda+1)$
4	$L_{yoz0\_2} = L_1/3$	$L_{2g} = L_1/(\lambda+1)$	$C_{1g} = C_2 - 3C_1$	$L_{2g} = L_1/(\lambda+1)$	$C_{yoz1\_2} = C_2 - 2C_1$
3	$L_{yoz0\_1} = L_1/(2\lambda+3)$	$L_{2g} = L_1/(\lambda+1)$	$L_{1g} = L_1/(3\lambda+3)$	$L_{2g} = L_1/(\lambda+1)$	$L_{yoz1\_1} = L_1/(3\lambda+2)$
2	$L_{yoz0\_2} = L_1/3$	$L_{2g} = L_1/(\lambda+1)$	$C_{1g} = C_2 - 3C_1$	$L_{2g} = L_1/(\lambda+1)$	$C_{yoz1\_2} = C_2 - 2C_1$
1	$L_{z00\_2} = L_1/(\lambda+3)$	$L_{xoz0\_2} = L_1$	$L_{xoz0\_1} = L_1/(2\lambda+3)$	$L_{xoz0\_2} = L_1$	$L_{z01\_2} = L_1/(2\lambda+2)$

The theoretical values of grounded terms

	1	2	3	4	5
5	$L_{z10\_2} = 0.384\mu H$	$L_{xoz1\_2} = 0.623\mu H$	$L_{xoz1\_1} = 0.277\mu H$	$L_{xoz1\_2} = 0.623\mu H$	$L_{z11} = 0.303\mu H$
4	$L_{yoz0\_2} = 1.1\mu H$	$L_{2g} = 0.767\mu H$	$C_{1g} = 0.3nF$	$L_{2g} = 0.767\mu H$	$C_{yoz1\_2} = 1.3nF$
3	$L_{yoz0\_1} = 0.344\mu H$	$L_{2g} = 0.767\mu H$	$L_{1g} = 0.256\mu H$	$L_{2g} = 0.767\mu H$	$L_{yoz1\_1} = 0.277\mu H$
2	$L_{yoz0\_2} = 1.1\mu H$	$L_{2g} = 0.767\mu H$	$C_{1g} = 0.3nF$	$L_{2g} = 0.767\mu H$	$C_{yoz1\_2} = 1.3nF$
1	$L_{z00\_2} = 0.524\mu H$	$L_{xoz0\_2} = 3.3\mu H$	$L_{xoz0\_1} = 0.344\mu H$	$L_{xoz0\_2} = 3.3\mu H$	$L_{z01\_2} = 0.384\mu H$

Supplementary Table 6. Grounded terms for the finite circuit at layer 5

	1	2	3	4	5
5	$L_{\text{corner\_7}} = L_1/(2\lambda - 1)$	$L_{x11\_2} = L_1/(\lambda - 1)$	$L_{x11\_1} = L_1/(3\lambda - 1)$	$L_{x11\_2} = L_1/(\lambda - 1)$	$L_{\text{corner\_8}} = L_2/3$
4	$C_{y10\_2} = 2C_1$	$L_{xoy1\_2} = L_2$	$C_{xoy1\_3} = 2C_1 + C_2$	$L_{xoy1\_2} = L_2$	$C_{y11\_2} = C_1 + C_2$
3	$L_{y10\_1} = L_1/(2\lambda - 2)$	$L_{xoy1\_2} = L_2$	$L_{xoy1\_1} = L_1/(3\lambda - 2)$	$L_{xoy1\_2} = L_2$	$L_{y11\_1} = L_1/(3\lambda - 1)$
2	$C_{y10\_2} = 2C_1$	$L_{xoy1\_2} = L_2$	$C_{xoy1\_3} = 2C_1 + C_2$	$L_{xoy1\_2} = L_2$	$C_{y11\_2} = C_1 + C_2$
1	$L_{\text{corner\_5}} = L_1/(\lambda - 2)$	$C_{y01\_2} = C_1$ $L_{y01\_2} = L_1$	$L_{x10\_1} = L_1/(2\lambda - 2)$	$C_{y01\_2} = C_1$ $L_{y01\_2} = L_1$	$L_{\text{corner\_6}} = L_1/(2\lambda - 1)$

The theoretical values of grounded terms

	1	2	3	4	5
5	$L_{\text{corner\_7}} = 0.589\mu H$	$L_{x11\_2} = 1.435\mu H$	$L_{x11\_1} = 0.371\mu H$	$L_{x11\_2} = 1.435\mu H$	$L_{\text{corner\_8}} = 0.333\mu H$
4	$C_{y10\_2} = 2nF$	$L_{xoy1\_2} = 1\mu H$	$C_{xoy1\_3} = 5.3nF$	$L_{xoy1\_2} = 1\mu H$	$C_{y11\_2} = 4.3nF$
3	$L_{y10\_1} = 0.717\mu H$	$L_{xoy1\_2} = 1\mu H$	$L_{xoy1\_1} = 0.418\mu H$	$L_{xoy1\_2} = 1\mu H$	$L_{y11\_1} = 0.371\mu H$
2	$C_{y10\_2} = 6.6nF$	$L_{xoy1\_2} = 1\mu H$	$C_{xoy1\_3} = 5.3nF$	$L_{xoy1\_2} = 1\mu H$	$C_{y11\_2} = 4.3nF$
1	$L_{\text{corner\_5}} = 2.538\mu H)$	$C_{y01\_2} = 1nF$ $L_{y01\_2} = 3.3\mu H$	$L_{x10\_1} = 0.717\mu H$	$C_{y01\_2} = 1nF$ $L_{y01\_2} = 3.3\mu H$	$L_{\text{corner\_6}} = 0.589\mu H$

In circuit experiments, traditional SMA connectors are typically secured to coaxial cables through threaded connections. However, due to the limited space between layers in the circuit model of this work (intended to minimize parasitic inductance and maintain aesthetics), threaded connections can be cumbersome in practice. Therefore, IPEX antenna bases were soldered onto the circuit board, and IPEX-to-SMA adapter cables were used to connect to the coaxial cables. The S_{11} parameter of each terminal was measured, and the corresponding self-impedance can be calculated using the formula

$$Z_{\text{in}} = Z_0 \cdot \frac{1 + S_{11}}{1 - S_{11}}. \tag{43}$$

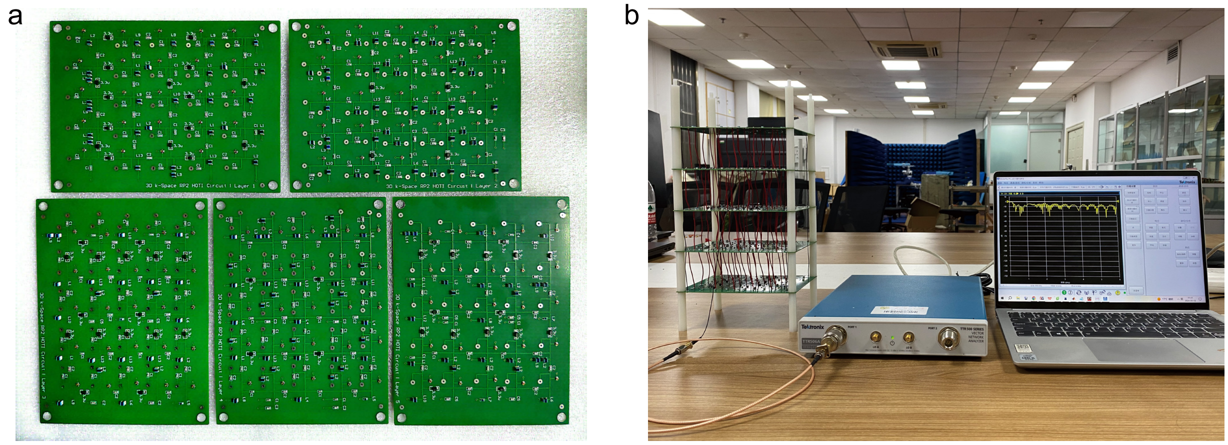

Supplementary Figure S9. **a** Overview of 5-layer circuit boards. **b** The experimental measurement platform.

[1] Chen ZY, Yang SA and Zhao YX. Brillouin klein bottle from artificial gauge fields. *Nat Commun* 2022; **13**: 2215.
